# Regulation of Smoothened Phosphorylation and High-Level Hedgehog Signaling Activity by a Plasma Membrane Associated Kinase

**DOI:** 10.1371/journal.pbio.1002481

**Published:** 2016-06-09

**Authors:** Shuangxi Li, Shuang Li, Yuhong Han, Chao Tong, Bing Wang, Yongbin Chen, Jin Jiang

**Affiliations:** 1 Department of Molecular Biology, University of Texas Southwestern Medical Center at Dallas, Dallas, Texas, United States of America; 2 Department of Developmental Biology, University of Texas Southwestern Medical Center at Dallas, Dallas, Texas, United States of America; 3 Kunming Institute of Zoology, Chinese Academy of Sciences, Kunming, Yunnan, China; 4 Department of Pharmacology, University of Texas Southwestern Medical Center at Dallas, Dallas, Texas, United States of America; University of Zurich, SWITZERLAND

## Abstract

Hedgehog (Hh) signaling controls embryonic development and adult tissue homeostasis through the G protein coupled receptor (GPCR)-family protein Smoothened (Smo). Upon stimulation, Smo accumulates on the cell surface in *Drosophila* or primary cilia in vertebrates, which is thought to be essential for its activation and function, but the underlying mechanisms remain poorly understood. Here we show that Hh stimulates the binding of Smo to a plasma membrane-associated kinase Gilgamesh (Gish)/CK1γ and that Gish fine-tunes Hh pathway activity by phosphorylating a Ser/Thr cluster (CL-II) in the juxtamembrane region of Smo carboxyl-terminal intracellular tail (C-tail). We find that CL-II phosphorylation is promoted by protein kinase A (PKA)-mediated phosphorylation of Smo C-tail and depends on cell surface localization of both Gish and Smo. Consistent with CL-II being critical for high-threshold Hh target gene expression, its phosphorylation appears to require higher levels of Hh or longer exposure to the same level of Hh than PKA-site phosphorylation on Smo. Furthermore, we find that vertebrate CK1γ is localized at the primary cilium to promote Smo phosphorylation and Sonic hedgehog (Shh) pathway activation. Our study reveals a conserved mechanism whereby Hh induces a change in Smo subcellular localization to promote its association with and activation by a plasma membrane localized kinase, and provides new insight into how Hh morphogen progressively activates Smo.

## Abstract 2

A study of both fruit flies and vertebrate cells shows that the plasma membrane-associated kinase Gish/CK1γ promotes transduction of the Hedgehog signal by phosphorylating Smoothened.

## Author Summary

The secreted glycoprotein Hedgehog (Hh) plays a conserved role in embryonic development and adult tissue homeostasis in species ranging from *Drosophila* to humans. Deregulation of Hh signal transduction contributes to a wide range of human disorders, including birth defects and cancer. The seven-transmembrane protein Smoothened (Smo) is an obligatory and conserved Hh signal transducer, but how Hh stimulates its activity remains unclear. Here we identify a plasma membrane associated kinase, Gilgamesh (Gish)/CK1γ, as a positive regulator of the Hh signaling activity. We find that Gish activates Hh signaling by phosphorylating a specific site in the Smo C-terminal intracellular tail. Phosphorylation of Smo by Gish is required for maximal activation of Smo and depends on membrane association of Gish and prior phosphorylation of Smo by protein kinase A (PKA). We also find that Hh stimulates the association of Smo with Gish after it travels to the plasma membrane, thus facilitating its phosphorylation by Gish. Finally, we provide evidence that CK1γ is found at the primary cilium in mammals and phosphorylates Smo to activate the Hh pathway. Our results uncover a conserved role of Gish/CK1γ in the regulation of Smo phosphorylation and provide new insight into the molecular underpinning of how Hh signal is transduced across the plasma membrane.

## Introduction

Hedgehog (Hh) signaling plays an essential role in embryonic development and adult tissue homeostasis, and its deregulation has been implicated in congenital diseases and cancers [1–6]. Hh exerts its biological influence through an intracellular signal transduction cascade that emanates from a G protein coupled receptor (GPCR)-family protein Smoothened (Smo) and culminates in the activation of the latent transcription factor Cubitus interruptus (Ci)/Glioma-associated oncogene homologue (Gli) [1,3,7,8]. In the signaling off state, Smo is inhibited by a twelve-transmembrane protein Patched (Ptc). Binding of Hh to Ptc alleviates such inhibition, allowing Smo to be phosphorylated and accumulate on the cell surface in *Drosophila* or primary cilia in vertebrates, where Smo adopts an open and active conformation to relay the Hh signal to the intracellular signaling components [9–15].

Phosphorylation plays a critical role in the regulation of Smo conformation and subcellular localization [16]. In *Drosophila*, unphosphorylated or hypophosphorylated Smo adopts a closed, inactive conformation [12] and is ubiquitinated and removed from the cell surface by both proteasome- and lysosome-mediated degradation [17,18]. Upon Hh stimulation, Smo is phosphorylated by protein kinase A (PKA) and casein kinase 1 (CK1), mainly the CK1α/ε isoforms, at three clusters of Ser/Thr residues in its carboxyl-terminal intracellular tail (C-tail) [19–22], which drives a conformational switch of Smo C-tail from the closed inactive to an open active conformation, leading to dimerization/oligomerization of the C-tail [12]. In addition, PKA/CK1α/ε-mediated phosphorylation inhibits ubiquitination of Smo, leading to its cell surface accumulation and activation [17,18]. In addition to PKA and CK1α/ε, Smo activity is also modulated by casein kinase 2 (CK2), atypical protein kinase C (aPKC), and G protein coupled receptor kinase 2 (Gprk2) [23–26]. Gprk2 promotes Smo activity by directly binding and phosphorylating the Smo C-tail to stabilize its active conformation [24]. In mammals, Hh-stimulated phosphorylation of Smo by CK1α and Gprk2 promotes its ciliary localization and active conformation [15]. Cell surface/ciliary accumulation of Smo is thought to be essential for its activation and function [14,27]; however, the underlying mechanism is still poorly understood.

Hh functions as a morphogen to specify different developmental outcomes in a concentration-dependent manner [1,3]. As such, Hh signal transduction needs to be tightly controlled to achieve pathway activities appropriate with the ligand inputs. Although the characterized phosphorylation events contribute to Smo activation, they do not represent all the activation mechanisms, as phospho-mimetic mutations failed to fully activate Smo in both *Drosophila* and mammals [15,19,24], suggesting that additional mechanisms, either phosphorylation-dependent or independent, may exist. Indeed, *Drosophila* Smo is phosphorylated at more than 26 Serine (Ser)/Threonine (Thr) residues, many of which have not been well characterized [20].

CK1γ, which is encoded by *gilgamesh* (*gish*) in *Drosophila* [28], is a membrane-associated Ser/Thr kinase of the CK1 family [29]. Gish/CK1γ has been implicated in the regulation of Wingless (Wg)/Wnt signaling by phosphorylating the co-receptor Arrow (Arr)/LRP5/6 [30,31]. Gish is also involved in glial cell migration in *Drosophila* eye [28], olfactory learning [32], and planar cell polarity (PCP)-mediated morphogenesis [33]. Mammals have three CK1γ isoforms encoded by different genes [29], making it difficult to study the role of CK1γ in development. In this study, we identified Gish as a positive regulator of Hh signaling through a genetic modifier screen. We demonstrated that Hh stimulates the association between Gish and Smo in a manner depending on cell surface localization of both Gish and Smo as well as PKA-mediated phosphorylation of Smo C-tail. We provided evidence that Gish phosphorylates a membrane proximal region of Smo C-tail to promote high levels of Hh pathway activity. However, loss of Gish only caused a minor defect in Hh pathway activity, likely due to a redundancy with another kinase(s). We also found that vertebrate CK1γ is localized at primary cilia depending on its membrane association, and that CK1γ promotes Smo phosphorylation and Sonic hedgehog (Shh) pathway activation depending on the primary cilia. Our results suggest that plasma membrane/ciliary-localized CK1γ plays a conserved role in Hh signaling by promoting the maximal levels of Smo activity.

## Results

### A Genetic Modifier Screen Identified Gish as a Positive Regulator of the Hh Pathway

**Fig 1:**
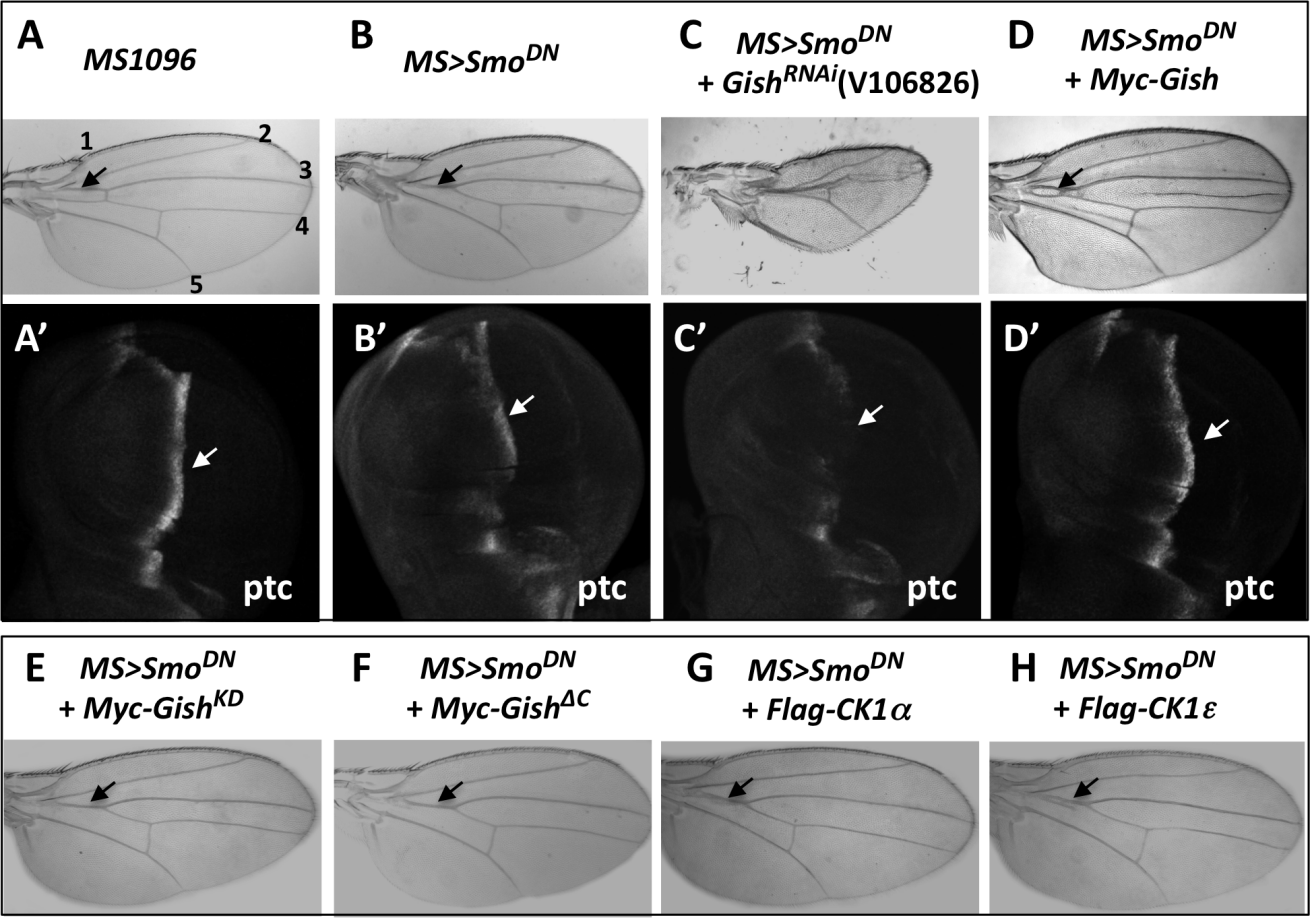
Gish positively regulates Hh signaling. (A–H) Control wing (A) or wings expressing a dominant negative Smo using *MS1096* (*MS>Smo*^*DN*^) either alone (B) or together with *UAS-Gish-RNAi* (C), *UAS-Myc-Gish* (D), *UAS-Myc-Gish*^*KD*^ (E), *UAS-Gish*^*ΔC*^ (F), *UAS-Flag-CK1α* (G), or *UAS-Flag-CK1ε* (H). *MS>Smo*^*DN*^ caused a fusion between veins 3 and 4 (arrow in B), which was enhanced by Gish RNAi (C) but partially suppressed by Gish overexpression (arrow in D). Overexpression of Gish^KD^, Gish^*Δ*C^, CK1*α*, or CK1ε failed to suppress the “fused wing” phenotype caused by *MS>Smo*^*DN*^. (A'-D') Late third instar wing discs of the indicated genotypes immunostained with an anti-Ptc antibody. *MS>Smo*^*DN*^ diminished *ptc* expression near the A/P boundary (arrow in B'). Gish RNAi further reduced while Gish overexpression restored *ptc* expression (arrows in C', D'). In this and the following figures, images are representatives of five or more adult wings or wing discs for each genotype.

To identify additional Hh pathway regulators, we have conducted an RNAi-based genetic modifier screen to identify enhancers or suppressors of a "fused wing" phenotype caused by expression of a dominant negative Smo (Smo^-PKA12^/Smo^DN^) with a wing-specific Gal4 drivers *MS1096* (*MS>Smo*^*DN*^) [19,24,25]. We found that expressing a *UAS-RNAi* line (V106826) targeting Gish, the *Drosophila* homologue of mammalian CK1γ, enhanced the “fused wing” phenotype caused by *MS>Smo*^*DN*^ (Fig 1A–1C). Using immunostaining with a Gish antibody, we found that Gish RNAi effectively knocked down Gish protein expression in wing imaginal discs (S1A–S1B' Fig). Moreover, two additional *UAS-Gish*^*RNAi*^ lines, V26003 and BL28066, enhanced the *MS>Smo*^*DN*^-induced phenotype in a similar fashion (S1C–S1F Fig). On the other hand, overexpression of a wild-type Gish, but not a kinase dead form (Gish^KD^), partially suppressed the “fused wing” phenotype caused by *MS>Smo*^*DN*^ (Fig 1D and 1E and S1K and S1L Fig).

Gish/CK1γ is localized to the plasma membrane due to its C-terminal palmitoylation [30,33]. Interestingly, overexpression of a soluble form of Gish with its palmitoylation site deleted, GishΔC [33], failed to rescue *MS>Smo*^*DN*^-induced wing phenotype, even though it was expressed at levels similar to the wild-type Gish (Fig 1F and S1M Fig compared with S1K Fig), suggesting that plasma membrane association of Gish is critical for its function in this context. Consistent with this notion, overexpression of other soluble CK1 family members, including CK1α and CK1ε, also failed to rescue the *MS>SmoDN* phenotype (Fig 1G and 1H).

To determine whether loss- or gain-of-Gish function modified the "fused wing" phenotype through the Hh pathway, we examined the expression of an Hh target gene ptc. In control late third instar wing imaginal discs, Hh induced ptc expression in A-compartment cells near the A/P boundary (Fig 1A'). In MS>Smo^DN^ wing discs, ptc expression near the A/P boundary was greatly reduced (Fig 1B'). Gish RNAi in MS>Smo^DN^ wing discs nearly abolished ptc expression near the A/P boundary in the wing pouch region where *MS1096* was expressed (Fig 1C'). On the other hand, overexpression of Gish restored *ptc* expression close to wild-type levels (Fig 1D'). Hence, gain- or loss-of-Gish activity can modulate Hh pathway activity.

To determine where Gish acts in the Hh pathway, we examined its genetic interaction with Fused (Fu), a Ser/Thr kinase acting downstream of Smo [11,34,35]. Inactivation of Fu by expressing a *USA-RNAi* transgene with *MS1096* (*MS>Fu*^*RNAi*^) caused a similar "fused wing" phenotype, albeit more severe than that caused by *MS>*Smo^DN^ (S1H Fig). However, neither Gish overexpression nor RNAi modified the wing phenotype caused by MS>Fu^RNAi^ (S1I and S1J Fig), suggesting that Gish may act upstream of Fu in the Hh pathway.

### Characterization of *gish* Mutants

**Fig 2:**
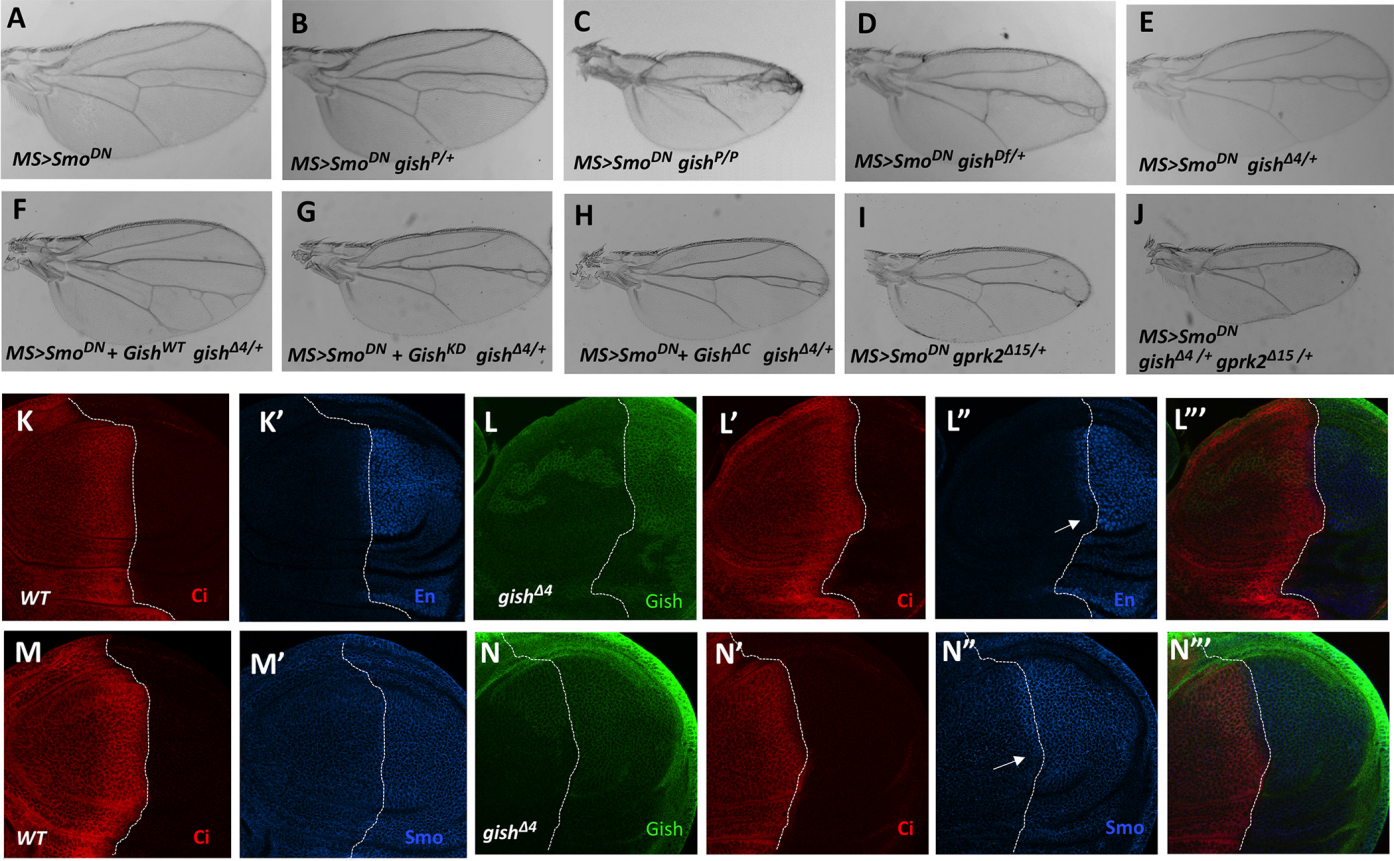
*gish* is required for high levels of Hh pathway activity and genetically interacts with *gprk2*. (A–J) Adult wings of the indicated genotypes. *MS>Smo*^*DN*^ caused a fusion between veins 3 and 4 (A). *MS>Smo*^*DN*^ wing carrying *gish*^*P*^ clones exhibited an enhanced phenotype (C). Heterozygosity for *gish*^*Df*^ (D) or *gish*^*Δ4*^ (E) but not *gish*^*P*^ (B) enhanced the fused wing phenotype caused by *MS>Smo*^*DN*^. Overexpression of wild-type Gish (F) but not Gish^KD^ (G) or Gish^*Δ*C^ (H) rescued the fused wing phenotype caused by *MS>Smo*^*DN*^ in *gish* heterozygous background. Heterozygosity for *gprk2*^*Δ15*^ also enhanced the *MS>Smo*^*DN*^ phenotype (I). *gprk2*^*Δ15*^ and *gish*^*Δ4*^ double heterozygosity enhanced the *MS>Smo*^*DN*^ phenotype more dramatically than either *gprk2*^*Δ15*^ or *gish*^*Δ4*^ single heterozygosity (J compared with E and H). (K–N‴) Late third instar wild type wing discs (K, K', M, M') or wing discs carrying *gish*^*Δ4*^ mutant clones induced at 24–48 h after egg laying (AEL) (L–L‴, N–N'') were immunostained to show the expression of Ci (red), Gish (green), En (blue in K', L", L‴), or Smo (blue in M', N", N‴). *gish*^*Δ4*^ mutant cells abutting the A/P boundary (demarcated by dashed lines) exhibited reduced En and Smo staining (arrows in L", N").

To confirm the Gish RNAi phenotype, we turned to *gish* mutants. *gish*^*KG03891*^ is a P-element insertion mutation and a strong allele of *gish* [32], which is referred to as *gish*^*P*^ hereafter. Although *gish*^*P*^ heterozygosity did not modify the fused wing phenotype caused by *MS>Smo*^*DN*^ (Fig 2B compared with Fig 2A), *MS>Smo*^*DN*^ wings carrying *gish*^*P*^ homozygous clones exhibited a greatly enhanced phenotype similar to that caused by Gish RNAi in *MS>Smo*^*DN*^ wings (Fig 2C compared with Fig 1C and S1E and S1F Fig). Interestingly, we found that heterozygosity for *gish* deficiency, *Df(3R)ED10639* (BL#9481), also enhanced the fused wing phenotype caused by *MS>Smo*^*DN*^ (Fig 2D), suggesting that *gish*^*P*^ is not a null allele. To obtain a *gish* null allele, we generated imprecise excision lines from *gish*^*P*^ and found several lines including *gish*^*Δ4*^ that could enhance the *MS>Smo*^*DN*^ phenotype similarly to *gish*^*Df*^ (Fig 2E). Consistent with *gish*^*Δ4*^ being a null allele, *gish*^*Δ4*^ mutant clones in wing discs exhibited diminished Gish immunostaining (S2A and S2B Fig). Furthermore, the enhancement of the *MS>Smo*^*DN*^ phenotype by *gish*^*Δ4/+*^ was reversed by coexpression of the wild-type Gish but not by coexpression of either Gish^KD^ or Gish^ΔC^ (Fig 2F–2H).

Our previous study identified Gprk2 as a positive regulator of Hh signaling in a similar genetic modifier screen [24]. Heterozygosity of a *gprk2* null allele, *gprk2*^*Δ15*^, also enhanced the fused wing phenotype caused by *MS>Smo*^*DN*^ (Fig 2I). Interestingly, taking away one copy of *gish* in this background (*MS>Smo*^*DN*^ *gish* ^*Δ4/+*^ *gprk2* ^*Δ*15/+)^ further enhanced the wing phenotype (Fig 2J). This dosage-sensitive genetic interaction between Gish and Gprk2 suggests that they may act in close proximity in the Hh signaling pathway.

### Gish Is Required for High-Level Hh Pathway Activity

We then induced *gish*^*Δ4*^ clones using the MARCM system [36] at 24–48 h or 48–72 h after egg laying (AEL). *gish*^*Δ4*^ clones (marked by green fluorescent protein [GFP] expression) induced at 48–72 h AEL survived to late third instar larval stages but did not affect the expression of Hh target genes *ptc* and *engrailed* (*en*) when localized in A-compartment cells near the A/P boundary (S2D–S2D" Fig and Fig 2F–2F" compared with S2C–S2C" and S2E–S2E" Fig). *gish*^*Δ4*^ clones induced at 24–48 h AEL were barely recovered, suggesting that *gish* mutant cells had a growth disadvantage and were competed out by wild-type cells over time. To recover early-induced clones, we generated *gish*^*Δ4*^ clones in a *Minute* background, which gave *gish* mutant cells a growth advantage [37]. As shown in Fig 2 and S2 Fig, *gish*^*Δ4*^ clones (marked by the lack of Gish staining) induced at 24–48 h AEL in the *Minute* background occupied large areas in late third instar wing discs (Fig 2L and 2N, S2H Fig). *ptc* expression, which is induced by intermediate levels of Hh, was not affected in *gish*^*Δ4*^ clones (S2H" and S2H‴ Fig); however, *en* expression in A-compartment cells near the A/P boundary, which is induced by peak levels of Hh, was diminished in *gish*^*Δ4*^ clones (arrow in Fig 2L–2L‴). In addition, Hh-induced Smo accumulation appeared to be attenuated in *gish*^*Δ4*^ clones (arrow in Fig 2N–2N‴). These results suggest that Gish is required for high levels of Hh signaling activity and may regulate the Hh pathway at the level of Smo.

### Gish Phosphorylates the Membrane Proximal Region of Smo C-tail

**Fig 3:**
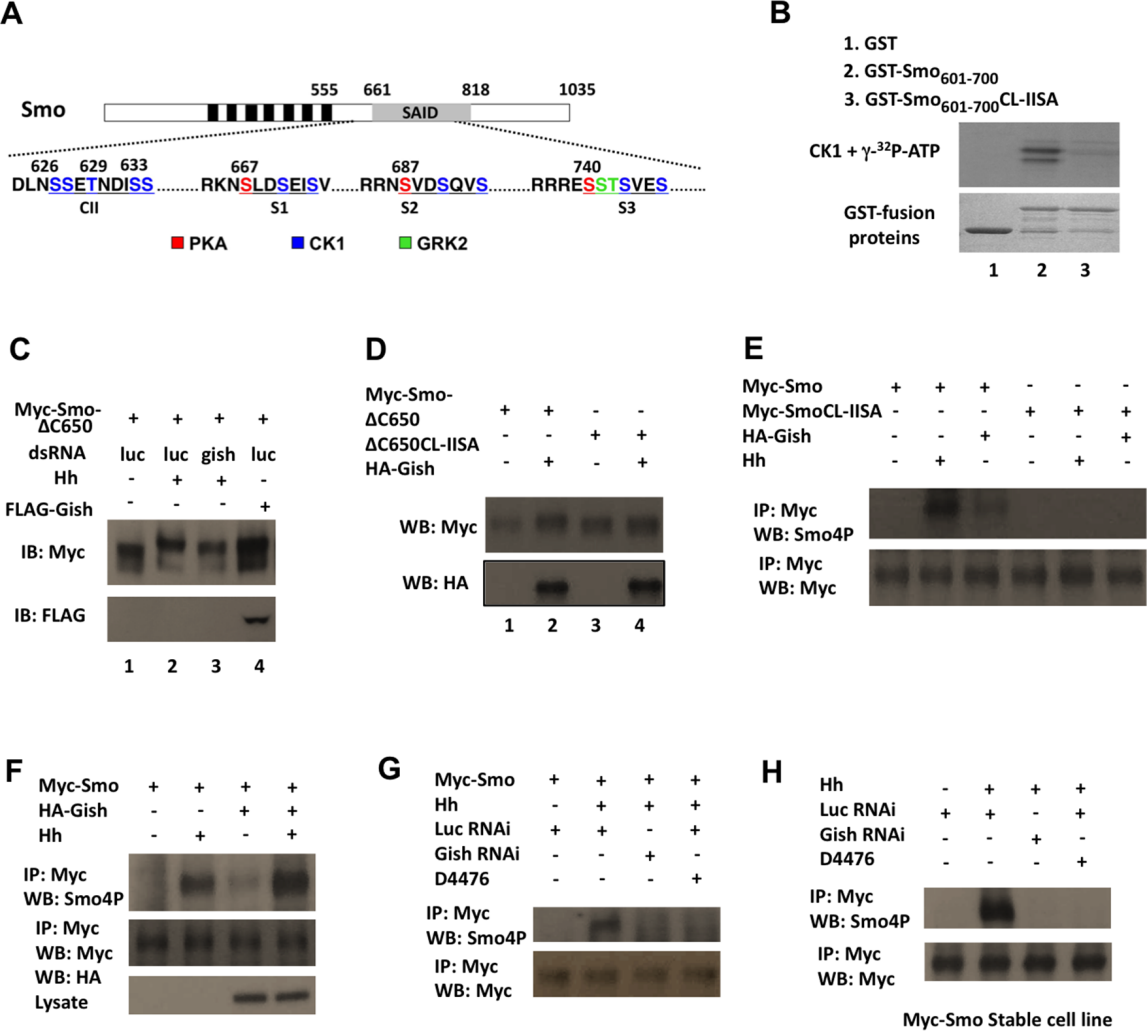
Hh promotes CL-II phosphorylation through Gish. (A) Diagram of Smo with PKA, CK1, and Gprk2 sites indicated by different colors. Black and gray boxes denote the seven transmembrane domains and the Smo auto-inhibited domain (SAID), respectively. (B) In vitro kinase assay using recombinant CK1 and GST-Smo fusion proteins carrying wild-type (GST-Smo_601-700_) or mutated CL-II site (GST-Smo_601-700_CL-IISA). (C, D) Western blots of lysates from S2 cells transfected with the indicated constructs, treated with or without the indicated dsRNA and Hh-conditioned medium. (E, F, L) Western blots of immunoprecipitation experiments from lysates of S2 cells transfected with the indicated constructs. Cells were grown in the presence or absence of Hh stimulation for 24 h and exposed to MG132 (50 μM) for 4 h before harvest. (G, H) S2 cells transiently (G) or stably (H) expressing Myc-Smo were treated with control RNAi, Gish RNAi, or D4476. Cells were grown in the presence or absence of Hh stimulation for 24 h and exposed to MG132 (50 μM) for 4 h, followed by immunoprecipitation and western blot analysis.

A previous study revealed that Smo derived from Hh-stimulated S2 cells was phosphorylated at 26 Ser/Thr residues in its C-tail, including the three PKA/CK1 phosphorylation clusters and Gprk2 sites (Fig 3A) [20]. However, the kinases responsible for phosphorylating other sites, most notably, a membrane proximal cluster (CL-II) of Ser/Thr residues, _623_DlNSSETNDISS_634_ (underlined S/T residues were phosphorylated sites detected by Mass Spec) [20], have not been identified (Fig 3A). A close inspection of the CL-II site indicates that it contains Ser/Thr residues falling into the consensus sites for the CK1 family kinases: D/E/(p)S/T(X)_1-3_S/T, in which the underlined S/T is the CK1 site, whereas X represents any amino acid [29]. Indeed, a Glutathione S-transferase (GST) fusion protein containing the intact (GST-Smo_601-700_) but not the mutated CL-II site (GST-Smo_601-700_ CL-II SA) was phosphorylated by a recombinant CK1 in an in vitro kinase assay (Fig 3B). To determine whether CL-II is phosphorylated by Gish, we transfected S2 cells with a Smo construct, Myc-Smo△C650, which contains the CL-II site but lacks distal phosphorylation sites such as the PKA/CK1 phosphorylation clusters and Gprk2 sites. We found that Hh stimulation induced a mobility shift of Myc-Smo△C650, which is indicative of Smo phosphorylation [19], and that Hh-induced mobility shift of Myc-Smo△C650 was diminished by Gish RNAi (Fig 3C). Coexpression of Myc-Smo△C650 with Gish also induced a mobility shift of Myc-Smo△C650 (Fig 3C and 3D). Furthermore, mutating the CL-II site in Myc-Smo△C650 (Myc-Smo△C650CL-IISA) abolished its mobility shift induced by either Hh stimulation or Gish overexpression (Fig 3D). Taken together, these results suggest that Hh induces phosphorylation of CL-II through Gish.

### Hh Stimulates CL-II Phosphorylation by Plasma Membrane-Associated Gish

To characterize CL-II phosphorylation in the context of full-length Smo, we generated a phospho-specific antibody named Smo4P using the phospho-peptide NDLN(_P_S)(_P_S)E(_P_T)NDI(_P_S)STW as an antigen (see Materials and Methods). Western blot analysis indicated that purified Smo4P antibody recognized GST-Smo_601-700_ but not GST-Smo_601-700_CL-llSA after in vitro phosphorylation by CK1. We found that Smo4P recognized Myc-Smo but not Myc-SmoCL-llSA derived from S2 cells stimulated with Hh or coexpressing Flag-Gish (Fig 3E and 3F). Overexpression of Gish further increased the Hh-stimulated Smo4P signal (Fig 3F). To further demonstrate that Hh stimulates CL-II phosphorylation through Gish, we examined the phosphorylation state of Myc-Smo derived from transiently transfected S2 cells or a stably expressing cell line treated with control or Gish dsRNA and stimulated with Hh. We found that Gish RNAi abolished Hh-stimulated Smo4P signal associated with Myc-Smo (Fig 3G and 3H). A recent study argued that Gprk2 is responsible for CL-II phosphorylation [38]; however, we found that Gprk2 RNAi or overexpression did not affect Smo4P signal intensity associated with Myc-Smo (S3A and S3B Fig). Furthermore, treatment of Myc-Smo-expressing cells with a pharmacological CK1 inhibitor, D4476, also abolished Hh-stimulated Smo4P signal (Fig 3G and 3H). These results suggest that Hh stimulates CL-II phosphorylation through Gish rather than Gprk2.

**Fig 4:**
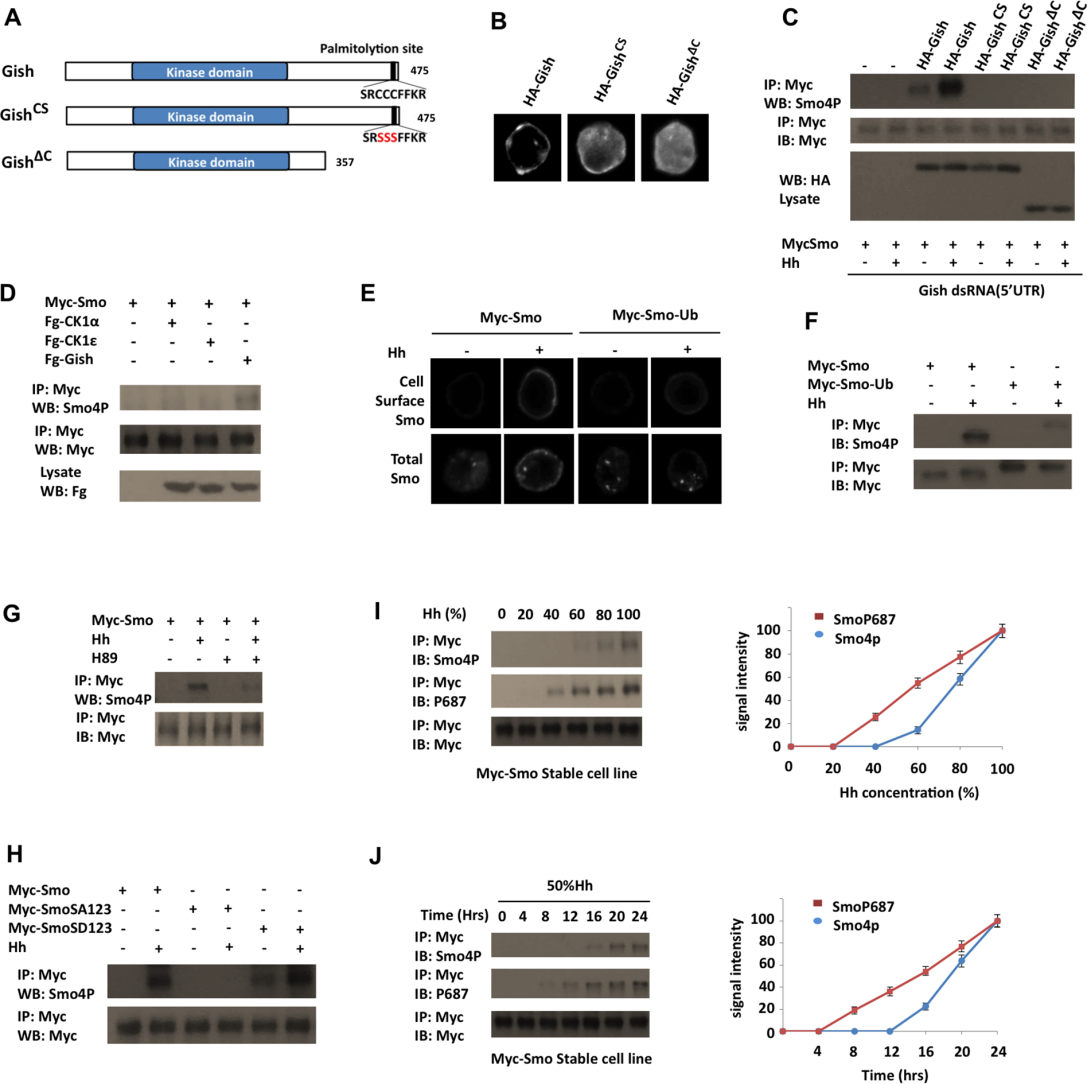
CL-II phosphorylation depends on PKA and cell surface localization of both Smo and Gish. (A) Diagrams of wild-type Gish and its mutant derivatives. (B) Immunostaining of S2 cells transfected with the indicated Gish constructs. (C) Western blots of immunoprecipitation experiments from lysates of S2 cells treated with dsRNA targeting the *gish* 5’ UTR and transfected with Myc-Smo together with the indicated Gish constructs. Cells were grown in the presence or absence of Hh stimulation for 24 h and exposed to MG132 (50 μM) for 4 h before harvest. (E, F) S2 cells were transfected with Myc-Smo or Myc-Smo-Ub and stimulated with or without Hh, followed by immunostaining to visualize cell surface Smo (top panels in E) or total Smo (bottom panels in E), or by immunoprecipitation and western blot analysis with the indicated antibodies. (G) S2 cells stably expressing Myc-Smo were treated with or without Hh-conditioned medium and H89 and exposed to MG132 (50 μM) for 4 h, followed by immunoprecipitation and western blot analysis by the indicated antibodies. (H) Western blots of immunoprecipitation experiments from lysates of S2 cells transfected in indicated Smo constructs. Cells were grown in the presence or absence of Hh stimulation for 24 h and exposed to MG132 (50 μM) for 4 h before harvest. (I, J) S2 cells stably expressing Myc-Smo were treated with different levels of Hh or the same level of Hh for different periods of time, followed by immunoprecipitation and western blot analysis by the indicated antibodies. Quantification of Smo4P and P687 signals is shown to the right. The signal intensities at 100% Hh or 24 h are defined as 100. Data are means ± SD from three independent experiments.

Gish/CK1γ is attached to the inner leaf of the plasma membrane through its C-terminal palmitoylation (Fig 4A) [30]. Indeed, HA-Gish was mainly associated with cell membrane when expressed in S2 cells (Fig 4B). By contrast, HA-Gish^CS^ and HA-Gish^ΔC^, which have their C-terminal palmitoylation signal (SRCCCFFKR) substituted (SRSSSFFKR) or deleted, respectively (Fig 4A), exhibited cytoplasmic distribution (Fig 4B) [33]. We then coexpressed HA-Gish, HA-Gish^CS^, or HA-Gish^ΔC^ with Myc-Smo in S2 cells with endogenous Gish knocked down by dsRNA targeting the 5' UTR of *gish*. Western blot analysis with Smo4P indicated that only the membrane-associated form of Gish (HA-Gish) but not the cytosolic variants (HA-Gish^CS^ and HA-Gish^ΔC^) could support CL-II phosphorylation in response to Hh (Fig 4C), suggesting that plasma membrane association of Gish is critical for CL-II phosphorylation. In addition, we found that coexpression of the cytosolic CK1 family members CK1α and CK1ε with Myc-Smo did not significantly increase the Smo4P signal in either the presence or absence of Hh stimulation (Fig 4D and S3C Fig). These results may explain why overexpression of wild-type Gish but not Gish^ΔC^, CK1α, or CK1ε could partially rescue the wing phenotype caused by *MS-Smo*^*DN*^ (Fig 1D and 1F–1H).

### CL-II Phosphorylation Depends on Cell Surface Localization of Smo

To determine whether cell surface localization of Smo is important for CL-II phosphorylation, we employed a Myc-tagged Smo variant that has a ubiquitin (Ub) moiety fused to its C-terminus (Myc-Smo-Ub) [18]. We confirmed the previous finding that Myc-Smo-Ub failed to accumulate on the cell surface in response to Hh (Fig 4E) [18,39]. However, Myc-Smo-Ub could still be phosphorylated by PKA and accumulate on the cell surface in response to PKA phosphorylation (S4 Fig), suggesting that adding the Ub moiety to the Smo C-terminus did not cause an overall structure change of Smo C-tail to preclude its phosphorylation by any kinase. We then determined whether failure to accumulate on the cell surface affected Hh-induced phosphorylation of Smo at the CL-II site. As shown in Fig 4F, Hh-induced Smo4P signal associated with Myc-Smo-Ub was dramatically reduced compared to that associated with Myc-Smo. This result suggests that cell surface localization of Smo is critical for its phosphorylation by Gish.

### CL-II Phosphorylation Depends on PKA-Mediated Phosphorylation of Smo

Previous studies suggest that Hh stimulates Smo phosphorylation by PKA and CK1α/ε at three clusters of Ser/Thr residues in the middle region of Smo C-tail (Fig 3A), and that these phosphorylation events promote Smo cell surface accumulation and conformational change [12,19]. To determine whether PKA-mediated phosphorylation of the distal sites regulates Gish-mediated phosphorylation of the membrane proximal sites, we first treated Myc-Smo expressing cells with a pharmacological PKA inhibitor H89 and found that inhibition of PKA activity diminished the Hh-stimulated Smo4P signal associated with Myc-Smo (Fig 4G). We then compared CL-II phosphorylation of a PKA-phosphorylation deficient (Myc-SmoSA123) or a phospho-mimetic (Myc-SmoSD123) form of Smo with that of wild-type Myc-Smo [19]. We found that Hh failed to stimulate the Smo4P signal associated with Myc-SmoSA123 (Fig 4H). On the other hand, Myc-SmoSD123 exhibited enhanced basal Smo4P signal, which was further enhanced upon Hh stimulation (Fig 4H). Taken together, these results suggest that PKA/CK1-mediated phosphorylation of Smo in the distal region facilitates Gish-mediated phosphorylation of the juxtamembrane region of Smo C-tail.

### Differential Regulation of CL-II and PKA Site Phosphorylation by Hh

We next determined whether PKA site phosphorylation and CL-II phosphorylation were induced by different levels of Hh. S2 cells stably expressing Myc-Smo were treated with Hh-conditioned medium containing different levels of Hh-N (20%, 40%, 60%, 80%, or 100%) for 4 h. Cell lysates were immunoprecipitated with anti-Myc antibody, followed by western blot analysis with either anti-SmoP687, which recognized the phosphorylated PKA site (S687) as well as two downstream CK1 sites [40], or anti-Smo4P. As shown in Fig 4I, SmoP687 signal began to be detected at 40% Hh, whereas Smo4P signal was detected only when the Hh levels exceeded 60% Hh. Myc-Smo expressing S2 cells were also treated with 50% Hh for different periods of time (4, 8, 12, 16, 20, and 24 h). SmoP687 signal began to be detected 8 h after Hh stimulation, whereas Smo4P was not detected until 16 h after Hh stimulation (Fig 4J). Hence, the CL-II phosphorylation appears to require higher levels of Hh or longer exposure to the same level of Hh than the PKA-site phosphorylation.

### CL-II Phosphorylation Is Required for Optimal Smo Activity

**Fig 5:**
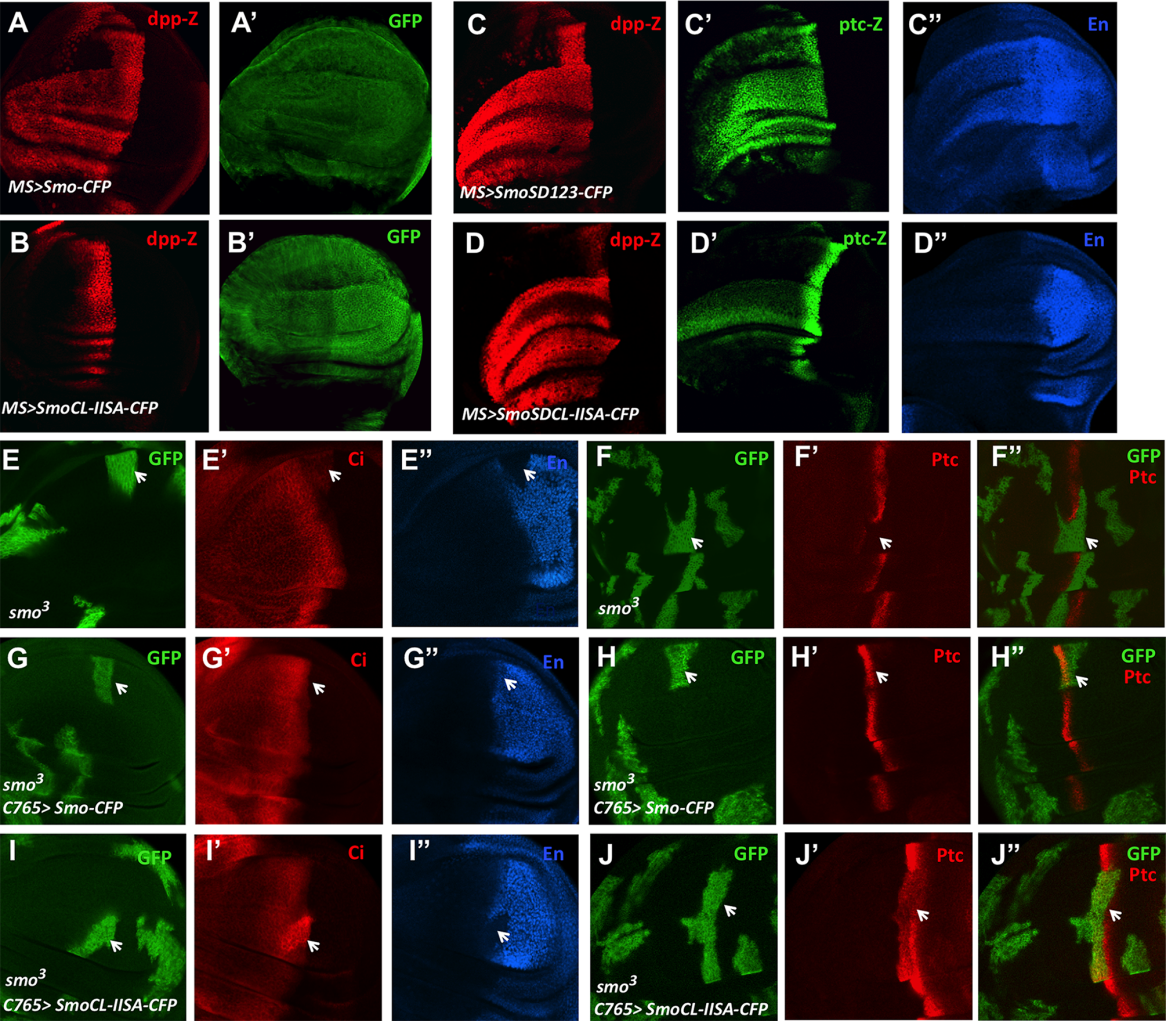
CL-II phosphorylation is required for optimal Smo activation. (A–D") Wing discs expressing *MS>Smo-CFP* (A–A′), *MS>SmoCL-IISA-CFP* (B, B’), *MS>SmoSD123-CFP* (C–C") or *MS>SmoSDCL-IISA-CFP* (D–D") were immunostained to show the expression of *dpp-lacZ*, GFP, *ptc-lacZ* or En. (E–J") Wing discs carrying *smo*^*3*^ MACRM clones in the absence (E–F") or presence of *C765>Smo-CFP* (G–H") or *C765>SmoSDCL-IISA-CFP* (I–J") were immunostained to show the expression of GFP, Ci, Ptc, and En. Arrows indicate *smo*^*3*^ clones (marked by GFP) near the A/P boundary.

To determine the functional importance of CL-II phosphorylation, we mutated CL-II in the context of Smo-cyan fluorescent protein (CFP) or SmoSD123-CFP to generate SmoCL-IISA-CFP and SmoSDCL-IISA-CFP, respectively (see Materials and Methods). Consistent with previous findings [11,19], overexpression of Smo-CFP using the *MS1096* Gal4 driver (*MS>Smo-CFP*) induced ectopic expression of *dpp-lacZ*, which is a low-threshold Hh target gene (Fig 5A); however, SmoCL-IISA-CFP failed to induce ectopic *dpp-lacZ* expression (Fig 5B). While *MS>SmoSD123-CFP* induced ectopic expression of not only *dpp-lacZ* but also *ptc-lacZ* and *en* at high levels (Fig 5C–5C"), SmoSDCL-IISA-CFP failed to induce ectopic expression of *en* and only induced ectopic expression of *ptc-lacZ* at low levels, although the ectopic expression of *dpp-lacZ* was not affected by the CL-IISA mutation (Fig 5D–5D"). Hence, mutating the CL-II site in Smo compromised its ability to activate the Hh pathway. Coexpression of Flag-Gish with Smo-CFP increased the ectopic *dpp-lacZ* expression, leading to more dramatic overgrowth of the wing discs (S5A and S5B' Fig). By contrast, coexpression of Flag-Gish with SmoCL-IISA-CFP did not alter its activity (S5C and S5D' Fig), consistent with the notion that Gish promotes Hh signaling activity through phosphorylating the CL-II site.

To discern the in vivo function of CL-II phosphorylation more precisely, we expressed Smo-CFP and SmoCL-IISA-CFP at low levels using the weak Gal4 driver *C765* (*C765>Smo*) in wing discs carrying *smo*^*3*^ mutant clones. Our previous study revealed that the levels of Smo derived from *C765>Smo* were only slightly higher than that of endogenous Smo [22]. We found that *C765>Smo-CFP* completely rescued the expression of both *ptc* and *en* in *smo*^*3*^ mutant clones located near the A/P boundary (Fig 5G–5H" compared with Fig 5E–5F"). By contrast, *C765>SmoCL-IISA-CFP* failed to rescue *en* expression and only partially rescued *ptc* expression in *smo*^*3*^ clones (Fig 5I–5J"). In addition, A/P boundary-located *smo*^*3*^ cells expressing *C765>SmoCL-IISA-CFP* accumulated high levels of full-length Ci (Fig 5I'), suggesting SmoCL-IISA-CFP could still inhibit Ci processing but fail to induce the maturation of full-length Ci into the active but labile form [41]. Furthermore, we found that SmoCL-IISA-CFP, like Smo-CFP, promoted Ci nuclear localization in *smo* mutant clones (S5E–S5F′′′ Fig). Previous studies revealed that Ci nuclear translocation occurs in A-compartment cells more than ten cells away from the A/P boundary, where there are low levels of Hh [42,43]. Collectively, these results demonstrate that CL-II phosphorylation is essential for Smo to transduce high levels of Hh signaling activity but is dispensable for low levels of Hh pathway activity.

### Gish- and Gprk2-Mediated Phosphorylation Promotes High Levels of Smo Activity

Our previous study demonstrated that Gprk2 promotes high levels of Hh pathway activity by regulating the active state of Smo through both kinase activity dependent and independent mechanisms [24]. Gprk2 phosphorylated Smo at Ser741/Thr742 and S1013/S1015, and mutating these sites (GPSA12) in the context of SmoSD123 (SmoSDGPSA) compromised Hh pathway activity [24]. However, expression of a Smo variant with the Gprk2 sites mutated (SmoGPSA) using the strong Gal4 driver *MS1096* fully rescued the expression of Hh target genes including *ptc* and *en* in A/P boundary-located smo^3^ clones. We reasoned that overexpression of SmoGPSA to levels much higher than the physiological level could mask its signaling defect. Indeed, expression of SmoGPSA with *C765* only rescued *ptc* expression but failed to restore *en* expression in A/P boundary-located *smo*^*3*^ clones (S6A–S6B′′ Fig). Hence, Grpk2-mediated phosphorylation of Smo is also required for its optimal activity.

**Fig 6:**
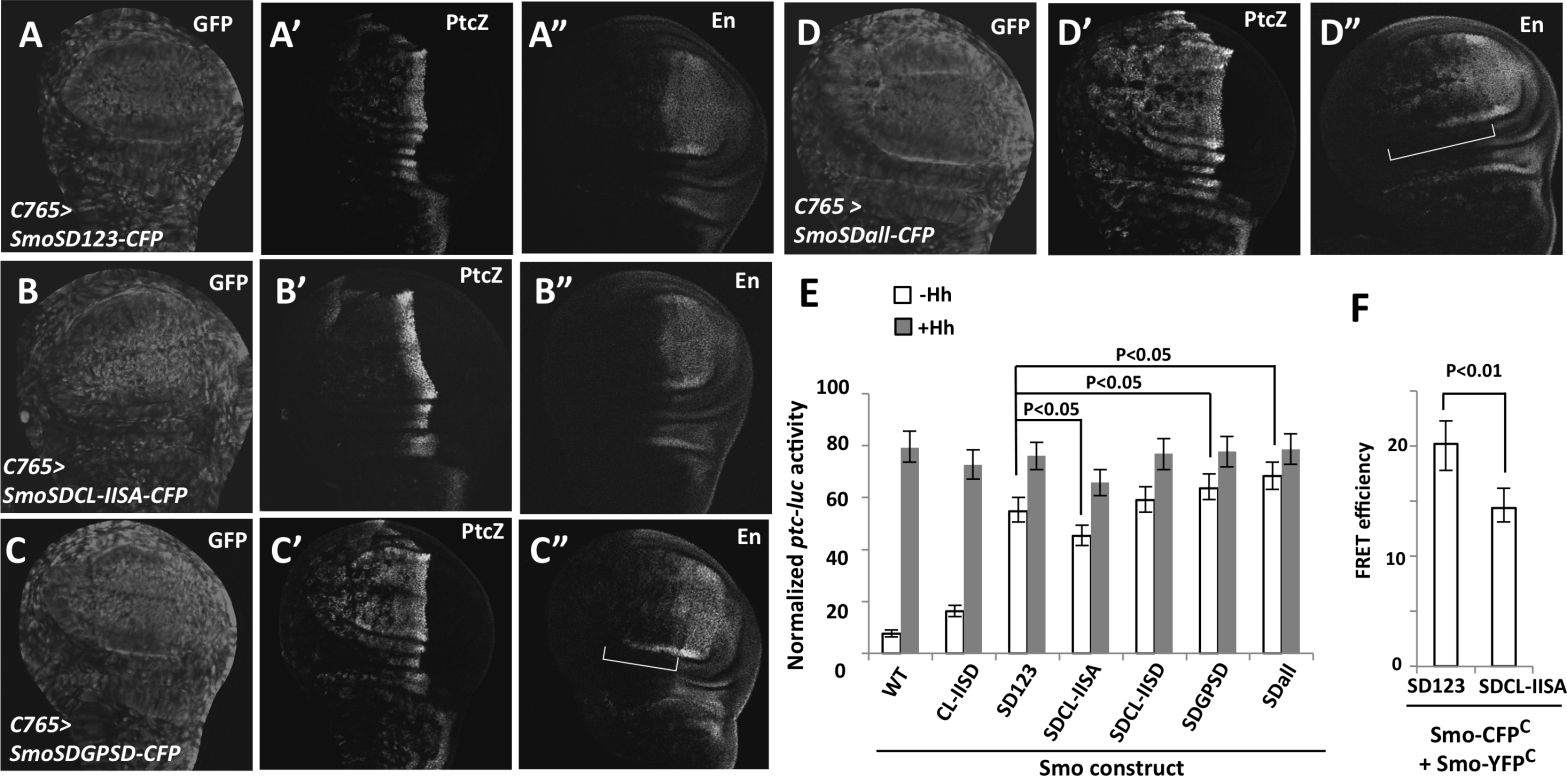
Graded Smo activity generated by phospho-mimetic mutations in PKA/CK1, Gprk2 and Gish phosphorylation sites. (A–D") Wing discs expressing the indicated Smo constructs with *C765* were immunostained to show the expression of GFP, *ptc-lacZ*, and En. *C765>SmoSDall-CFP* induced ectopic *en* expression in a broader domain of A-compartment cells than *C765>SmoSDGPSD-CFP* (indicated by the brackets in C", D"). (E) *ptc-luciferase* reporter assay in Cl8 cells expressing the indicated Smo constructs and treated with Hh-conditioned or control medium. Data are means ± SD of normalized *ptc-luc* activity from three independent experiments. *P*-values are Student's *t* tests. (F) FRET efficiency from SmoSD123-CFP^C^/SmoSD123-YFP^C^ and SmoSDCL-IISA-CFP^C^/ SmoSDCL-IISA-YFP^C^ expressed in S2 cells. Data are means ± SD, n = 10 cells. *P*-values are Student's *t* tests.

To further determine the contribution of Gish- and Gprk2-mediated phosphorylation of Smo to Hh pathway activation and how differential Smo phosphorylation generates degraded Hh pathway activity, we introduced phospho-mimetic mutations to the CL-II site (CL-IISD) in the context of SmoSDGPSD, which contains phospho-mimetic mutations in the three PKA/CK1 phosphorylation clusters (SD123) and two Gprk2 sites (GPSD) [24], to generate SmoSDall. Hence, SmoSDall represents Smo with the highest level of phosphorylation, followed by SmoSDGPSD, SmoSD123, and SmoSDCL-IISA. When expressed in wing discs using *C765*, SmoSD123 induced ectopic *ptc-lacZ* expression in A-compartment cells both distant from and close to the A/P boundary, albeit at levels lower than that of endogenous *ptc-lacZ* at the A/P boundary (Fig 6A and 6A'). In addition, SmoSD123 induced weak ectopic *en* expression in A-compartment cells near the A/P boundary (Fig 6A"). SmoSDCL-IISA induced ectopic *ptc-lacZ* expression at lower levels than SmoSD123 and failed to induce any ectopic *en* expression (Fig 6B–6B"), consistent with its activity being weaker than SmoSD123. Similar to SmoSDCL-IISA, SmoSDGPSA also exhibited weaker activity than SmoSD123 because it only induced low levels of ectopic *ptc* expression but failed to induce ectopic *en* expression (S6C–S6D′′ Fig). On the other hand, SmoSDGPSD and SmoSDall induced ectopic expression of *ptc-lacZ* and *en* at higher levels than SmoSD123 (Fig 6C–6D"). Although both SmoSDall and SmoSDGPSD induced ectopic *ptc-lacZ* expression at similar levels (Fig 6C' and 6D'), SmoSDall induced ectopic *en* expression in more anterior cells than SmoSDGPSD (Fig 6C" and 6D"). We also examined the activity of several Smo variants with different phospho-mimetic mutations in Clone 8 (Cl8) cells through a *ptc-luciferase* reporter assay. As shown in Fig 6E, the levels of ectopic Smo activity correlated with the levels of Smo phosphorylation. Hence, increasing levels of Smo phosphorylation progressively increased its signaling activity.

Our previous studies revealed that phosphorylation of Smo C-tail induced a conformational change resulting in its dimerization/oligomerization, as indicated by an increased fluorescence resonance energy transfer (FRET) between C-terminally tagged CFP and yellow fluorescent protein (YFP) [12,24]. We found that mutating the CL-II site in SmoSD123 reduced its C-terminal FRET (Fig 6F), suggesting that CL-II phosphorylation may promote the active Smo conformation.

### Smo and Gish Form a Complex Stimulated by Hh

**Fig 7:**
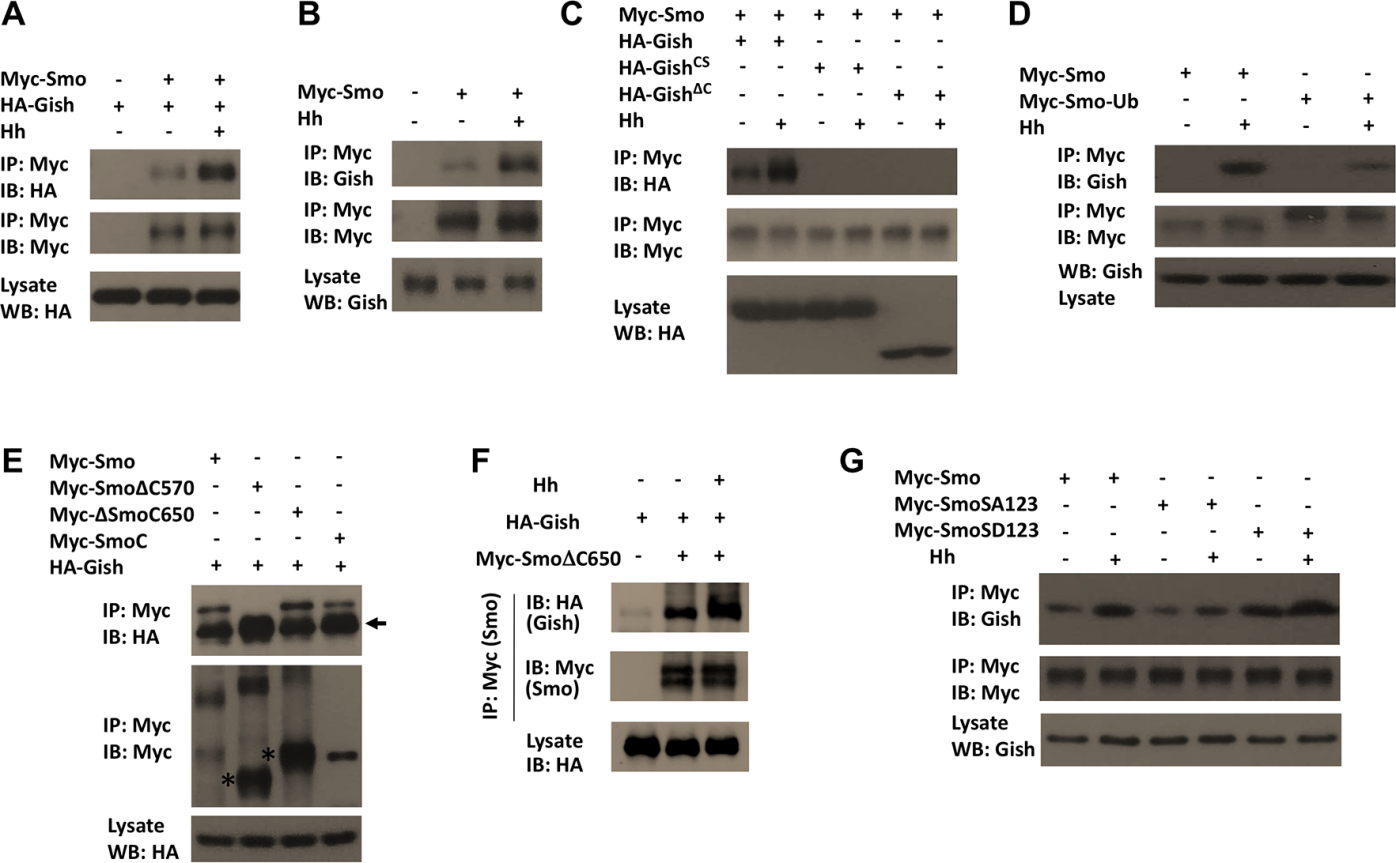
Gish forms a complex with Smo regulated by Hh and PKA. (A–G) Western blots of coimmunoprecipitation experiments from lysates of S2 cells transfected with the indicated constructs. Cells were grown in the presence or absence of Hh stimulation for 24 h. In A–D and G, cells were exposed to MG132 (50 μM) for 4 h before harvest. Myc-Smo coimmunoprecipitated with HA-Gish but not HA-Gish^CS^ or HA-Gish^CS^, and Smo/Gish association was enhanced by Hh stimulation (C). Preventing Smo cell surface accumulation by artificially conjugating an Ub moiety to the C-terminus of Smo (Myc-Smo-Ub) diminished its interaction with Gish (D). HA-Gish interacted with Myc-Smo, Myc-SmoΔ650, and Myc-SmoC but not with Myc- SmoΔ570 (E). Arrow indicates IgG and asterisks indicate monomeric forms of SmoΔ570 and SmoΔ650 (E). Hh stimulation increased the association between HA-Gish and Myc-SmoΔ650 (F). The SA mutation reduced while the SD mutation enhanced Smo/Gish association (G).

We next sought to determine the mechanism by which Hh stimulates Smo phosphorylation by Gish. Because Gish is associated with the plasma membrane, we speculated that Hh might stimulate the formation of a Smo-Gish complex at cell surface. Indeed, when expressed in S2 cells, Myc-Smo coimmunoprecipitated with endogenous Gish as well as coexpressed HA-Gish, and the amount of Gish in the Smo immunoprecipitates dramatically increased after Hh stimulation (Fig 7A and 7B). The formation of the Smo-Gish complex depends on the plasma membrane association of Gish, as Myc-Smo failed to pull down the cytosolic forms of Gish, HA-Gish^CS^, and HA-Gish^ΔC^ (Fig 7C). In addition, Myc-Smo-Ub pulled down much less Gish compared with Myc-Smo after Hh stimulation (Fig 7D), suggesting Hh-induced Smo cell surface localization of Smo is critical for its interaction with Gish.

When cotransfected into S2 cells, HA-Gish formed a complex with Myc-SmoCT (Smo C-tail) but failed to bind Myc-SmoΔC570, which lacks the C-tail (Fig 7E), suggesting that Smo interacts with Gish through its C-tail. HA-Gish was also associated with Myc-SmoΔC650 in S2 cells (Fig 7E). Furthermore, this association was enhanced upon Hh stimulation (Fig 7F), suggesting that Hh signaling facilitates the binding of Gish to the membrane proximal region of the Smo C-tail.

Finally, we determined whether Smo-Gish association is regulated by PKA-mediated phosphorylation of Smo. We found that mutating the PKA phosphorylation sites to Ala (SA123) attenuated Hh-stimulated Smo-Gish complex formation, whereas the phospho-mimetic mutations of PKA sites and adjacent CK1 sites (SD123) increased both the basal and Hh-stimulated Smo-Gish complex (Fig 7G). The influence of PKA phosphorylation of Smo on Smo-Gish interaction could explain why CL-II phosphorylation is affected by PKA (Fig 4G and 4H).

### Ciliary Localized CK1γ Regulates Smo Phosphorylation and Shh Signaling

**Fig 8:**
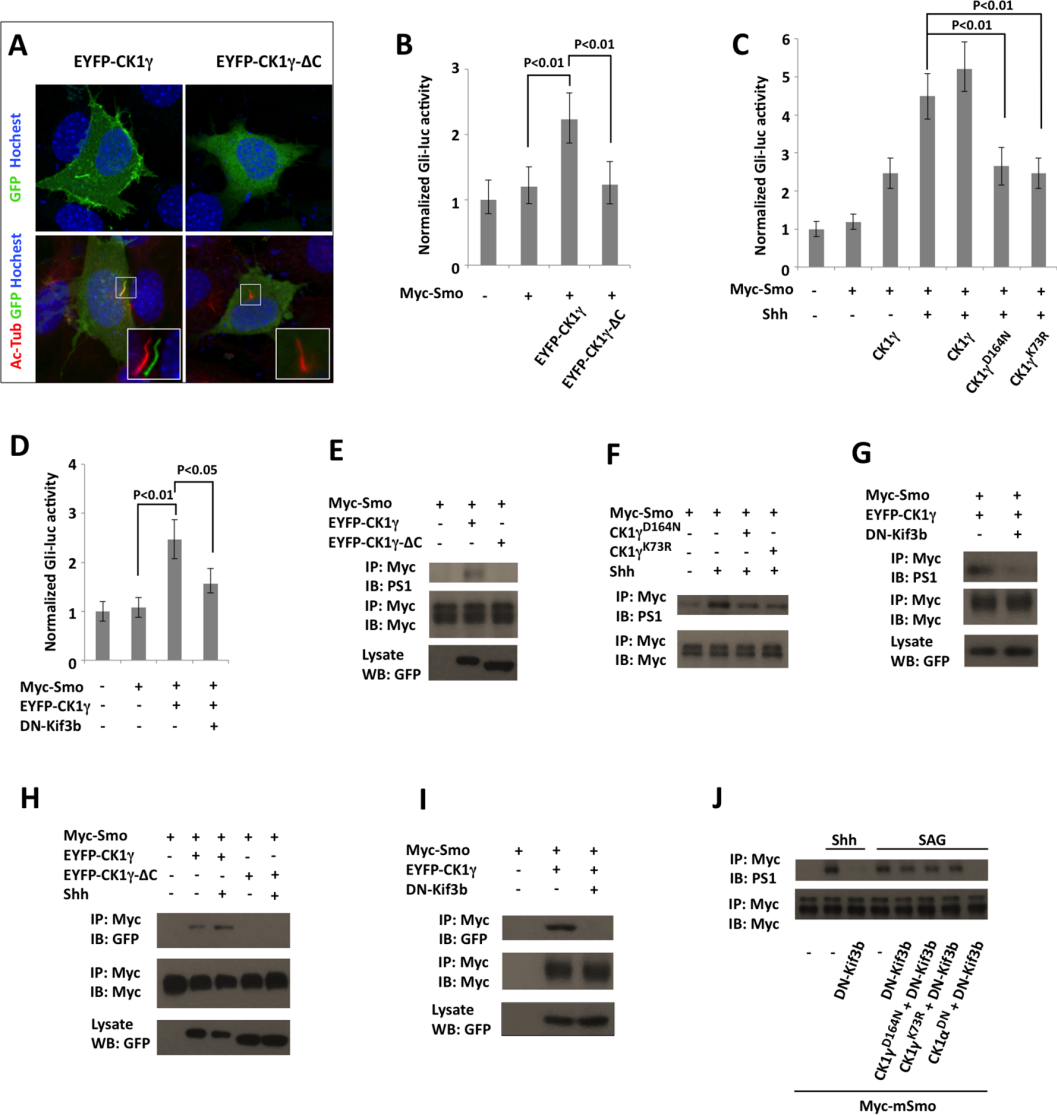
Cilium-localized CK1γ promotes Smo phosphorylation and Shh pathway activity. (A) NIH3T3 transfected with EYFP-CK1γ or EYFP-CK1γ-ΔC were immunostained with antibodies against acetylated tubulin (Ac-Tub), GFP, and Hoechst. EYFP-CK1γ but not EYFP-CK1γ-ΔC was localized on the primary cilium. (B–D) *Gli-luciferase* assay in NIH3T3 cells transfected Myc-Smo and the indicated CK1γ expression constructs in the presence or absence of DN-Kif3b, and treated with or without Shh. Data are means ± SD of normalized *Gli-luc* activity from three independent experiments. *P*-values are Student's *t* tests. (E–G) NIH3T3 cells were transfected Myc-Smo and the indicated CK1γ expression constructs in the presence or absence of DN-Kif3b, and treated with or without Shh. Cell lysates were immunoprecipitated with Myc antibody followed by western blot analysis with Myc and PS1 antibodies. (H, I) NIH3T3 cells were transfected with Myc-Smo and the indicated CK1γ expression constructs in the presence or absence of DN-Kif3b and treated with or without Shh. Cell lysates were immunoprecipitated with Myc antibody followed by western blot analysis with GFP and Myc antibodies. (J) NIH3T3 cells were transfected with the indicated constructs and treated with or without Shh or SAG, followed by immunoprecipitation and western blot analysis with the indicated antibodies.

We next determine whether CK1γ regulates Shh signaling in mammalian cells. When expressed in NIH3T3 cells, an enhanced yellow fluorescent protein (EYFP)-tagged Xenopus CK1γ (EYFP-CK1γ) was accumulated on the plasma membrane as well as on the primary cilium, whereas a cytosolic form of CK1γ, EYFP-CK1γ-ΔC, which lacks the C-terminal palmitoylation site [30], failed to localize on the primary cilium (Fig 8A), suggesting that ciliary localization of CK1γ depends on its plasma-membrane association. In a *Gli-luciferase* (*Gli-luc*) reporter assay, EYFP-CK1γ but not EYFP-CK1γ-ΔC stimulated Shh pathway activity (Fig 8B). By contrast, two dominant-negative forms of CK1γ CK1γ^K73R^ and CK1γ^D164N^, which specifically inhibited CK1γ activity [30], suppressed Shh-stimulated *Gli-luc* reporter activity (Fig 8C). Our previous study revealed that Shh stimulated the phosphorylation of mammalian Smo C-tail at multiple clusters of Ser/Thr residues, including a membrane-proximal cluster (S1) important for Smo activation [15]. Western blot analysis using an antibody (PS1), which recognizes phosphorylated S1 site [15], indicated that overexpression of EYFP-CK1γ but not EYFP-CK1γ-ΔC could stimulate Smo phosphorylation at the S1 site (Fig 8E). On the other hand, overexpression of either CK1γ^K73R^ or CK1γ^D164N^ attenuated Shh-stimulated S1 phosphorylation (Fig 8F). We found that EYFP-CK1γ and Myc-Smo formed a complex when coexpressed in NIH3T3 cells and that CK1γ/Smo association was enhanced upon Shh stimulation (Fig 8H). By contrast, EYFP-CK1γ-ΔC failed to interact with Smo even in the presence of Shh (Fig 8H). Because EYFP-CK1γ-ΔC failed to accumulate on the primary cilium, we speculated that the function of CK1γ in the regulation of Smo phosphorylation and Shh pathway activity might depend on the primary cilium. To disrupt the primary cilium, we overexpressed a dominant negative form of Kif3b (DN-Kif3b), a subunit of the Kinesin-II motor required for the cilium formation [15,44]. Indeed, DN-Kif3b overexpression blocked CK1γYFP/Myc-Smo association (Fig 8I), CK1γ-stimulated S1 phosphorylation (Fig 8G), and *Gli-luc* reporter activity (Fig 8D), suggesting that the primary cilium is required for CK1γ to bind and phosphorylate Smo.

A recent study revealed that the cell-membrane-permeable Smo agonist SAG could activate Smo without its ciliary accumulation [45]. Indeed, we found that SAG stimulated Smo phosphorylation at the S1 site in NIH3T3 cells transfected with DN-Kif3b, although Shh failed to stimulate Smo phosphorylation in these cells (Fig 8J), suggesting that SAG can induce Smo phosphorylation in the absence of the primary cilia, albeit at lower efficiency than in the presence of the primary cilia. This cilium-independent Smo phosphorylation was abolished when cells were transfected with a dominant negative form of CK1α (CK1α^DN^ [46]); however, the dominant negative forms of CK1γ (CK1γ^K73R^ and CK1γ^D164N^) failed to block cilium-independent Smo phosphorylation induced by SAG (Fig 8J). These results further support the notion that CK1γ regulates Smo phosphorylation in the primary cilium.

## Discussion

**Fig 9:**
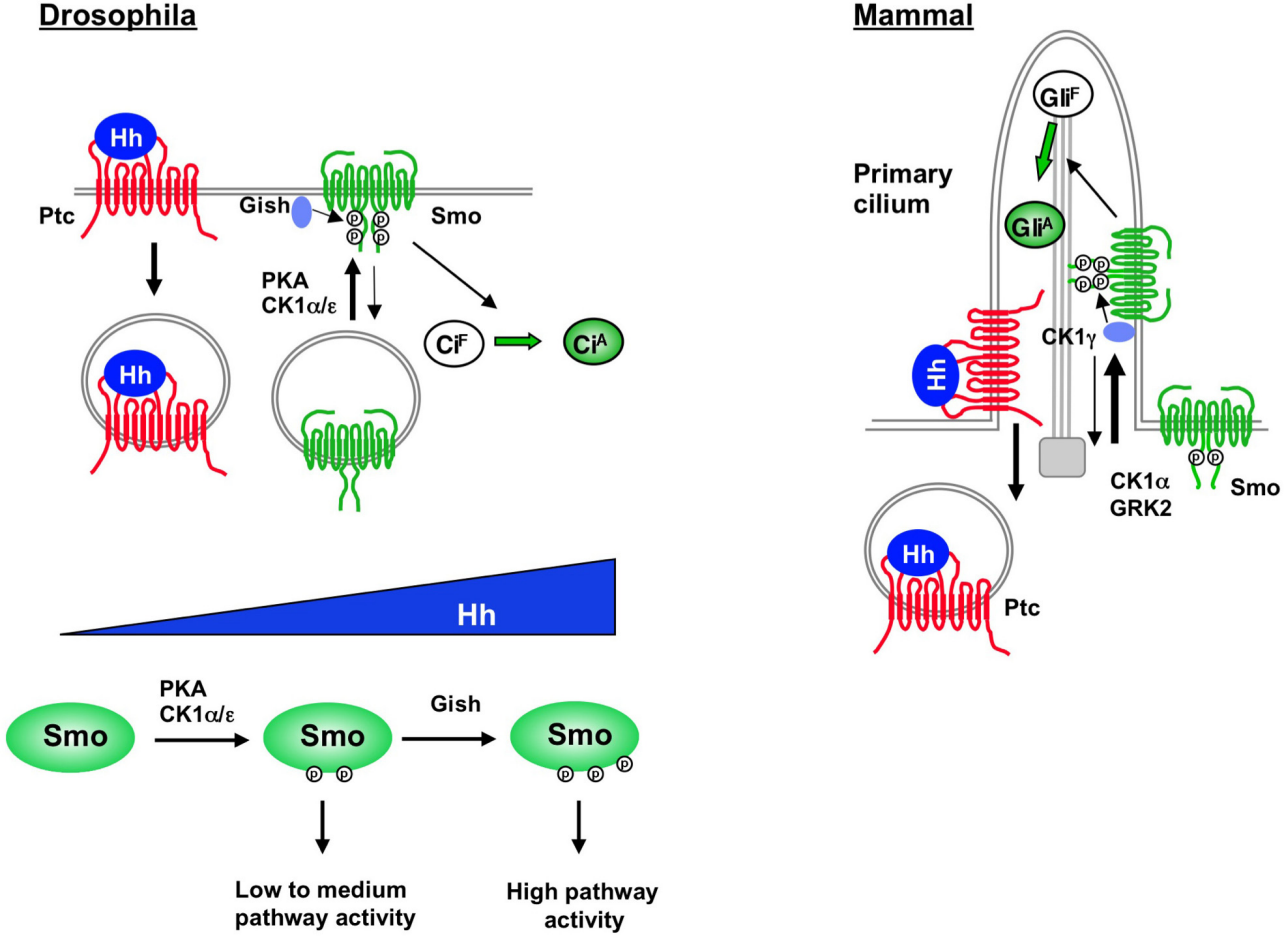
Plasma membrane/cilium-localized Gish/CK1γ modulates Hh signaling by phosphorylating Smo. Hh stimulates cell surface/ciliary accumulation of Smo, which facilitates its association, phosphorylation, and activation by Gish/CK1γ. In *Drosophila*, Hh morphogen gradient generates increasing levels of Smo activity by differential regulation of PKA/CK1- and Gish-mediated phosphorylation of Smo. See text for details.

In this study, we identified a plasma membrane-associated kinase Gish/CK1γ as a conserved positive regulator of the Hh pathway. We found that Hh stimulated the binding of Gish/CK1γ to Smo to phosphorylate a Ser/Thr cluster located in the membrane-proximal region of the Smo C-tail (CL-II site in *Drosophila* Smo and S1 site in mammalian Smo). We demonstrated that CL-II phosphorylation is required for the optimal Smo activation and Hh pathway activity. Interestingly, we found that Gish/CK1γ regulates Smo phosphorylation and Hh pathway activity depending on its plasma membrane/ciliary localization. We propose that cell surface/ciliary accumulation of Smo, which is facilitated by Hh stimulation, promotes its association, phosphorylation, and activation by Gish/CK1γ (Fig 9).

As an obligatory transducer of the Hh signal, Smo relays the positional information imposed by Hh morphogen gradient to different levels of pathway activity that elicit distinct developmental outcomes. How Smo is differentially activated by Hh morphogen gradient is incompletely understood; however, it has been suggested that different levels of Hh induce different levels of Smo phosphorylation, which are in turn translated into different levels of Hh pathway activity [16]. Indeed, it has been observed that increasing levels of Hh resulted in a progressive increase in the overall levels of Smo phosphorylation, and that increasing the number of phospho-mimetic mutation in the Smo C-tail led to a gradual increase in Hh pathway activity [11,12,15,40]. A previous study revealed that, in cultured *Drosophila* cells exposed to Hh, Smo was phosphorylated on at least 26 Ser/Thr residues in its C-tail [20]. While the distal phosphorylation sites including the three PKA/CK1 phosphorylation clusters have been well characterized, the biological function of and the kinases involved in the phosphorylation of the membrane-proximal sites, most notably the CL-II site, have remained largely unexplored. In addition, it remains unclear whether phosphorylation of CL-II is constitutive or stimulated by Hh because its phosphorylation state was not determined in the absence of Hh stimulation.

In this study, we generated an antibody (Smo4P) that recognized phosphorylated CL-II and demonstrated that CL-II phosphorylation was stimulated by Hh. Moreover, we found that CL-II phosphorylation was mediated by Gish in a manner depending on its membrane association. Both *gish* mutation and phosphorylation deficient CL-II mutation affected the expression of only high-threshold Hh target genes such as *en*, suggesting that Gish-mediated CL-II phosphorylation is not absolutely required for Hh signal transduction but rather plays a modulatory function in fine-tuning Hh signaling strength. We noticed that the reduction of Hh pathway activity in *gish* mutant clones was less severe compared with that associated with CL-II mutation (Fig 2, S2 Fig and Fig 5). One possibility is that we did not completely overcome the perdurance issue even when *gish* clones were generated at 24–48 h AEL. The early-expressed *gish* gene products could persist even after many rounds of cell division, and residual Gish kinase activity might still exist in *gish* mutant cells to partially phosphorylate CL-II. Indeed, we didn't observe any change in Hh pathway target gene expression in *gish* mutant clones generated at 48–72 h AEL, many of which are >30 cells in size, suggesting that *gish* gene products generated at early time points survived many rounds of cell divisions. Another possibility is that in the absence of Gish or when Gish activity is compromised, another kinase(s)—for example, other members of CK1 family—may partially compensate for the loss of Gish, although these kinases do not phosphorylate CL-II as effectively as Gish.

A recent study suggested that CL-II phosphorylation was mediated by Gprk2 without providing direct evidence [38]. However, we found that CL-II phosphorylation was not affected by Gprk2 knockdown or overexpression (S3 Fig). In addition, a recombinant GRK failed to phosphorylate a Smo fragment containing the CL-II site in an in vitro kinase assay [24]. Instead, our previous study indicated that Gprk2 phosphorylated Smo at Ser/741/Thr742 (GPS1) and Ser1013/Ser1015 (GPS2) and that mutating these sites to nonphosphorable Ala in the SmoSD123 background attenuated the activity of this constitutively active form of Smo [24]. Here, we further demonstrated that Smo with Gprk2 site mutated (SmoGPSA) failed to activate *en* in *smo* mutant clones located near the A/P boundary (S6 Fig), suggesting that Gprk2-mediated Smo phosphorylation is also required for high-levels of Hh signaling activity. Interestingly, both Gish- and Gprk2-mediated phosphorylation was regulated by PKA (Fig 4) [24], which phosphorylated Smo at S667, S687, and S740 [19–21]. Indeed, mutating the three PKA sites to Ala (SmoSA123) diminished while converting the PKA sites and adjacent CK1 sites to phospho-mimetic residues (SmoSD123) promoted CL-II phosphorylation. Although SmoSD123 could activate both low- and high-threshold Hh target genes when expressed at high levels, expression of SmoSD123 at close to physiological levels resulted in low to intermediate levels of Hh pathway activity (Fig 6A–6A") [19]. Phospho-mimetic mutations at Gprk2 or both Gprk2 and Gish sites in the SmoSD123 background resulted in a progressive increase in its activity (Fig 6D-6D"). Hence, Hh-induced phosphorylation of Smo at PKA sites may confer low to medium levels of pathway activation, while high levels of pathway activity require further phosphorylation by Gish and Gprk2, which is "primed" by PKA-mediated phosphorylation. Consistent with CL-II being critical for high-threshold Hh target gene expression, its phosphorylation requires higher levels of Hh or longer exposure to the same levels of Hh than PKA site phosphorylation (Fig 4I and 4J), suggesting graded Hh signals may differentially regulate PKA- and Gish-mediated phosphorylation of Smo to progressively increase Smo activity (Fig 9).

How does PKA regulate CL-II phosphorylation? Our previous study revealed that Smo adopts a closed conformation in which its C-terminal region folds back and lies in close proximity to its third intracellular loop, whereas Hh-induced phosphorylation at PKA/CK1 sites promotes an open conformation [12]. It is possible that the Gish-binding pocket is masked when Smo C-tails adopt the closed conformation, whereas phosphorylation-mediated conformation switch exposes the Gish-binding pocket. Indeed, we found SmoSD123 exhibited high basal binding to Gish, whereas SmoSA123 lost Hh-stimulated binding to Gish (Fig 7G). However, Hh could further stimulate the binding of Gish to SmoSD123 and increase CL-II phosphorylation in SmoSD123 (Figs 4H and 7G), implying that Hh could regulate Smo/Gish association and CL-II phosphorylation through additional mechanism(s). We noticed that Hh increased the binding of Gish to SmoΔ650 (Fig 7F), which lacks the C-terminal region, including the three PKA sites, suggesting that Hh signaling may induce a conformation change in the transmembrane helixes of Smo, similar to those observed for GPCRs in response to agonist stimulation [47], to expose the juxtamembrane binding site for Gish.

Hh-induced phosphorylation of Smo by PKA promotes its cell surface accumulation [19], which may also contribute the elevated CL-II phosphorylation upon Hh stimulation. In this regard, it is interesting to note that Gish is associated with the plasma membrane through its C-terminal palmitoylation. Cell surface accumulation of Smo will render its close proximity with the plasma membrane-associated Gish and facilitate its binding to Gish. Indeed, Smo failed to bind cytosolic forms of Gish, Gish^CS^ and Gish^ΔC^, both of which were unable to phosphorylate the CL-II site in response to Hh stimulation. In addition, artificial conjugation of a ubiquitin moiety to Smo (Myc-Smo-Ub) prevented Hh-stimulated Smo cell surface accumulation, Smo-Gish association, and CL-II phosphorylation (Figs 4E, 4F and 7D).

Interestingly, we found that vertebrate CK1γ is localized to the primary cilium depending on its C-terminal palmitoylation. Deleting the palmitoylation site (CK1γ-ΔC) prevented its ciliary localization, its association with mammalian Smo (mSmo), and its ability to phosphorylate mSmo and promote Shh pathway activity. Furthermore, depleting primary cilia by expressing a dominant negative for of Kif3b (DN-Kif3b) also affected CK1γ Smo interaction and phosphorylation by CK1γ. Taken together, these results suggest that only ciliary localized but not the cytosolic CK1γ is capable of phosphorylating Smo to promote Shh pathway activation. As Shh induces mSmo ciliary accumulation, we propose that ciliary accumulation of mSmo may facilitate its binding to and phosphorylation by CK1γ, which contributes to optimal Smo activation (Fig 9). Because abnormal Smo activation contributes to many types of human cancer, the finding that CK1γ is a conserved positive regulator of Hh signaling raises an interesting possibility that interfering with the interaction between CK1γ and Smo may serve as a strategy for cancer treatment.

## Materials and Methods

### *Drosophila* Mutants and Transgenes

All flies were raised on standard yeast and molasses-based food at 25°C. Gal4 drivers used in this study are *MS1096* and *C765* [22,24,48]. *UAS* transgenes are: *UAS-Smo*^*-PKA12*^*/Smo*^*DN*^, *UAS-Smo*^*-PKA123*^*/SmoSA123*, and *UAS-SmoSD123* [19]; *UAS-Smo-CFP*, *UAS-SmoSD123-CFP*, *UAS-SmoSDGPSD-CFP*, and *UAS-Smo-GPS1A2A-Fg/SmoGPSA* [24]; *UAS-Myc-Gish* (BL#41764), *UAS-Myc-Gish*^*ΔC*^ (BL#41769), and *UAS-Myc-Gish*^*KD*^ (BL#41766); *UAS-Flag-CK1α* and *UAS-Flag-CK1ε* [31]; and *UAS-Gish*^*RNAi*^ (VDRC#106826, VDRC#26003, and BL#28066). Xenopus CK1γ DNA constructs are: *pCS-EYFP-CK1γ*, *pCS-EYFP-CK1γ -ΔC*, *pCS-CK1γ* ^*D164N*^, and *pCS-CK1γ* ^*K73R*^ [30]. Mutant flies are: *smo*^*3*^ [10], *gish*^*KG03891*^/*gish*^*P*^ (BL#13263), *Gprk2*^*Δ15*^ [24], *Df(3R)ED10639* (BL#9481). *gish*^*Δ4*^ was generated by imprecise excision of the P-element from *gish*^*KG03891*^. Gish^CS^, SmoCL-IISA (S626A, S627A, S633A, S634A), SmoCL-IISD (S626D, S627D, S633D, S634D), SmoSDCL-IISA, SmoSDCL-IISD, and SmoSDall were generated by PCR-based site-directed mutagenesis and confirmed by DNA sequencing. Mutant clones were generated by standard *FRT/FLP* mediated mitotic recombination or the MARCM system as previously described [36,49]. Mutant clones were generated using the following genotype: MARCM clones for *gish*^*Δ4*^ (*yw hs-FLP; tub-Gal4; FRT82B tubGal80/FRT82B gish*^*Δ4*^), *gish*^*Δ4*^ clones in *Minute* background (*yw hs-FLP; FRT82B M(3) hs-CD2/FRT82B gish*^*Δ4*^), *gish*^*P/P*^ wings expressing Smo^DN^ (*yw MS1096 UAS-Flp; UAS-Smo*^*-PKA12*^*; FRT82B M(3) hs-CD2/FRT82B gish*^*KG03891*^), *smo* clones with or without expressing *smo* transgenes (*yw hs-FLP UAS-GFP; tubGal80 FRT40/smo*^*3*^ *FRT40; tub-Gal4*, *yw hs-FLP; tubGal80 FRT40/smo*^*3*^ *FRT40; C765/UAS-Smo-CFP or SmoCL-IISA-CFP*, or *yw hs-FLP UAS-GFP; tubGal80 FRT40/smo*^*3*^ *FRT40; C765/ UAS-Smo-GPSA*).

### Cell Culture, Transfection, Immunostaining, Immunoprecipitation, and Western Blot and FRET Analysis

*Drosophila* S2 cells were cultured in *Drosophila* SFM (Invitrogen) with 10% fetal bovine serum, 100 U/ml of penicillin, and 100 mg/ml of streptomycin at 24°C. Transfections were carried out using the Calcium Phosphate Transfection Kit (Specialty Media). Hh-conditioned medium treatment was carried out as described [50]. Unless mentioned otherwise, Hh-conditioned medium was used at a 6:4 dilution ratio by fresh medium (referred to as 100% Hh). NIH3T3 cells were cultured in DMEM (Sigma-Aldrich) containing 10% bovine calf serum (ATCC) and were transfected using the GenJet Plus In Vitro DNA Transfection Kit (SignaGen). Immunostaining and western blot analyses were carried out using standard protocols as previously described [49,51]. For immunoprecipitation assay, S2 cells were harvested and washed twice with PBS after transfection for 48 h and then lysed on ice for 30 min with lysis buffer containing 1M Tris pH8.0, 5M NaCl, 1M NaF, 0.1M Na_3_VO_4_, 1% NP-40, 10% Glycerol, and 0.5M EDTA (pH8.0). Cell lysates were incubated with protein A–Sepharose beads (Thermo scientific) for 1 h at 4 C° to eliminate non-specific binding proteins. After removal of the protein-A beads by centrifugation, the cleared lysates were incubated with Myc (HA or Flag) antibody for 2 h or overnight. The complexes were collected by incubation with protein A–Sepharose beads for 1 h at 4 C°, followed by centrifugation. The immunoprecipitates were then washed three times for 5 min each with lysis buffer and were fractionated by SDS–PAGE. FRET analysis was carried out as previously described [12]. Antibodies used for this study are rat anti-Ci, 2A1 [52], mouse anti-Ptc (DSHB), mouse anti-En (DSHB), mouse anti-SmoN (DSHB), mouse anti-HA (Santa Cruz), rabbit anti-LacZ (Affinity Bioreagents), rabbit anti-GFP (Invitrogen), and mouse anti-Flag (Sigma). Rabbit anti-Smo4P antibody was generated by Abmart (http://www.ab-mart.com) using the synthetic phosphopeptide NDLN(PS)(PS)E(PT)NDI(PS)STW-C as antigen. The reactive antibody was purified by absorption on a phosphopeptide affinity column and was further purified by subtraction on a column containing a non-phosphopeptide DLNSSETNDISSTW-C. The resulting antibody was initially characterized by western blot analysis using GST-Smo_601-700_ and GST-Smo_601-700_CL-IISA.

### In Vitro Kinase Assay, Luciferase Assay, and RNAi in *Drosophila* Cultured Cells

In vitro kinase assay was performed as previously described [15]. Briefly, GST-fusion proteins were mixed with 0.1 mM ATP, 10 mCi γ-^32^p-ATP, and CK1δ (New England Biolabs) and incubated at 30°C for 1.5 h in reaction buffer. Phosphorylation of GST-fusion proteins was analyzed by autoradiography after SDS-PAGE. For *ptc-luc* reporter assays, Cl8 cells were transfected with 1 μg *ptc-luc* reporter construct and 50 ng RL-PolII renilla construct in 12 well plates together with 1 μg Smo constructs. After 48 h incubation, the reporter assays were performed using the Dual-Luciferase reporter assay system (Promega). Dual-Luciferase measurements were performed in triplicate using FLUOstar OPTIMA (BMG LABTECH). *Gli-luc* reporter assays were carried out as previously described [15]. Double-stranded (ds) RNA was generated by MEGAscript High Yield Transcription Kit (Ambion). Gish dsRNA targeting the coding region (amino acids 196–474) was generated by PCR using the primers 5′-GAATTAATACGACTCACTATA GGGAG AGGCAGAAC GTCAACAAAACGT-3 and 5′-GAATTAATACGACTCACTATAGGGA GATTTTTGGCGCGTCGATTTCTT-3′. Gish dsRNA targeting the 5' UTR was generated by PCR using the primers 5'-GAATTAATACGACTCACTATAGGGAGAAAAGTGTGTTTGTCAAATTGT-3' and 5'-GAATTAATACGACTCACTATAGGGAG ACTCACCGCCCACACTCACACG-3 '. Gprk2 dsRNA was generated by PCR using DNA template targeting Gprk2 amino acids 124–290 as described [24]. For the RNAi knockdown experiments, S2 cells were cultured in serum-free medium containing the indicated dsRNA for 8 h at 24°C. After adding FBS to a final concentration of 10%, dsRNA-treated cells were cultured for 24 h before transfection. Forty-eight hours after transfection, the cells were collected for analyses.

## Supporting Information

S1 Data Numerical data used in preparation of Figs 4I, 4J, 6E, 6F, 8B–8D and S3A. (XLSX)

S1 Fig Gish positively regulates Hh signaling upstream of Fu. (A–B') Gish expression visualized by immunostaining with a Gish antibody in a control wing disc (A, A') and a wing disc expressing UAS-Gish-RNAi by *ptc-Gal4* (B, B'). *dpp-lacZ* expression marks the A-compartment cells near the A/P boundary. (C–J) Adult wings of the indicated genotypes. Gish RNAi enhanced the *MS>Smo*^*DN*^ (E, F; compared with Fig 1B); however, neither Gish RNAi nor Gish overexpression modified the wing phenotype caused by Fu RNAi. (K–M) Wing discs expressing the indicated Myc-tagged Gish constructs were immunostained with a Myc antibody to show similar expression levels of Gish proteins.
(TIF)

S2 Fig Characterization of *gish*^*Δ4*^. (A–F") Wing discs carrying control or *gish*^*Δ4*^ clones generated by the MARCM system were immunostained for GFP (green), Gish (red in A', A", B', B"), Ptc (red in C', C", D', D"), Ci (red in E, E", F, F"), and En (blue in E', E", F', F"). Clones were induced at 48–72 h AEL and marked by GFP expression. Gish expression was abolished in *gish*^*Δ4*^ clones (B–B"). Neither *ptc* nor *en* expression was affected in *gish*^*Δ4*^ clones (arrows in D–D", F–F"). (G–H‴) A control wing disc (G, G') or wing disc carrying *gish*^*Δ4*^ clones generated in the Minute background at 24–48 h AEL were immunostained with Ci, Ptc, and Gish antibodies. *ptc* expression was not affected in *gish*^*Δ4*^ clones marked by the lack of Gish signal (arrows in H–H‴).
(TIF)

S3 Fig The CL-II site is phosphorylated by Gish but not by CK1α, CK1ε, or Gprk2. (A) S2 cells stably expressing Myc-Smo were treated with control or Gprk2 dsRNA and with or without Hh-conditioned medium, followed by immunoprecipitation and western blot analysis with the indicated antibodies. Western blot (middle panel) and RT-qPCR (right) experiments confirm Gprk2 knockdown efficiency.(B, C) Western blots of coimmunoprecipitation experiments from lysates of S2 cells transfected with Myc-Smo and the indicated CK1 or Gprk2 constructs. Cells were grown in the presence or absence of Hh stimulation for 24 h and exposed to MG132 (50 μM) for 4 h before harvesting.
(TIF)

S4 Fig Smo-Ub can be phosphorylated by PKA overexpression. (A) Western blot of coimmunoprecipitation experiments from lysates of S2 cells transfected with Myc-Smo or Myc-Smo-Ub together with mC*-YFP (a constitutively active form of PKA). Both Myc-Smo and Myc-Smo-Ub were phosphorylated by mC* at S687.(B) S2 cells were transfected with Myc-Smo or Myc-Smo-Ub either alone or together with mC*-YFP, followed by immunostaining to visualized cell surface Myc-Smo or Myc-Smo-Ub and mC*-CFP. Coexpression with the constitutively active form of PKA resulted in cell surface accumulation of both Myc-Smo and Myc-Smo-Ub.
(TIF)

S5 Fig Gish promotes Smo activity through the CL-II site. (A–D') *dpp-lacZ* and GFP expression in wing discs expressing the indicated Smo construct in the presence or absence Flag-Gish. Coexpression of Flag-Gish with Smo-CFP promoted its activity as indicated by more robust ectopic *dpp-lacZ* expression and enhanced overgrowth of the wing disc (B–B' compared with A–A'). Coexpression of Flag-Gish with SmoCL-IISA-CFP did not cause discernable change in the expression of *dpp-lacZ* or disc growth (D–D' compared with C–C'). (E–F‴) Wing discs carrying *smo*^*3*^ mutant clones expressing *C765>Smo-CFP* (E–E‴) or *C765>SmoCL-IISA-CFP* (F–F‴) were treated with 50 nM LMB for 2 h prior to immunostaining with GFP, Ci, and En antibodies. Both Smo-CFP and SmoCL-IISA-CFP promote Ci nuclear localization in *smo* mutant clones near the A/P boundary.
(TIF)

S6 Fig Gprk2 phosphorylation sites are required for optimal Smo activity. (A–B‴) Wing discs carrying *smo*^*3*^ mutant clones expressing *C765>SmoGPSA12* were immunostained to show the expression of GFP (green), which marks the *smo* mutant cells, Ci (red), and En (blue in A", A‴) or Ptc (blue in B", B‴). Expression of SmoGPS1A2A with *C765* rescued *ptc* but not *en* expression in *smo*^*3*^ mutant clone near the A-P boundary (arrowheads). (C–D'') Wing discs expressing *C765>SmoSD123-CFP* (C–C") or *C765>SmoSDGPSA-CFP* (D–D") were immunostained to show the expression of GFP, Ptc, and En. SmoSDGPSA exhibited reduced activity compared with SmoSD123.
(TIF)

## Abbreviations

AEL: after egg laying
aPKC: atypical protein kinase C
Arr: Arrow
CFP: Cyan fluorescent protein
Ci: Cubitus interruptus
CK1: casein kinase 1
CK2: casein kinase 2
Cl8: Clone 8
CL-II: cluster II
C-tail: carboxyl-terminal intracellular tail
EYFP: enhanced yellow fluorescent protein
En: Engrailed
FRET: Fluorescence resonance energy transfer
Fu: Fused
GFP: green fluorescent protein
Gish: Gilgamesh
Gli: Glioma-associated oncogene homologue
GPCR: G protein coupled receptor
Gprk2/GRK2: G protein coupled receptor kinase 2
GST: Glutathione S-transferase
Hh: Hedgehog
mSmo: mammalian Smo
PCP: planar cell polarity
PKA: protein kinase A
Ptc: Patched
SAID: Smo auto-inhibitory domain
Ser: Serine
Shh: Sonic hedgehog
Smo: Smoothened
Thr: Threonine
Ub: ubiquitin
Wg: Wingless
YFP: Yellow fluorescent protein
