# Supplementary figures and images for: Regulation of Smoothened Phosphorylation and High-Level Hedgehog Signaling Activity by a Plasma Membrane Associated Kinase
Source: PLoS Biol. 2016 Jun 9;14(6):e1002481. doi: 10.1371/journal.pbio.1002481 (PMC4900676; doi:10.1371/journal.pbio.1002481)

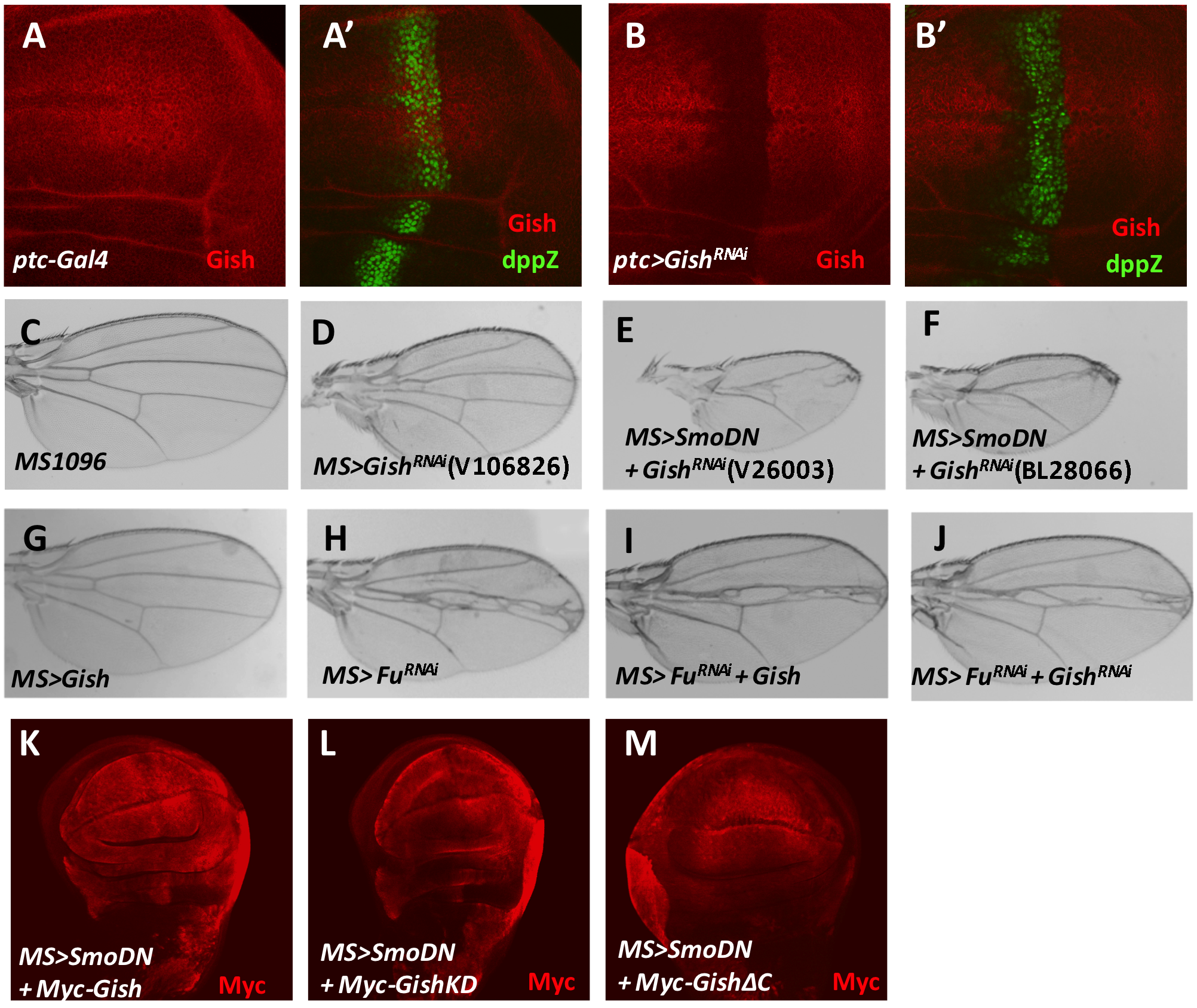

Supplement: S1 Fig — (A–B') Gish expression visualized by immunostaining with a Gish antibody in a control wing disc (A, A') and a wing disc expressing UAS-Gish-RNAi by ptc-Gal4 (B, B'). dpp-lacZ expression marks the A-compartment cells near the A/P boundary. (C–J) Adult wings of the indicated genotypes. Gish RNAi enhanced the MS>SmoDN (E, F; compared with Fig 1B); however, neither Gish RNAi nor Gish overexpression modified the wing phenotype caused by Fu RNAi. (K–M) Wing discs expressing the indicated Myc-tagged Gish constructs were immunostained with a Myc antibody to show similar expression levels of Gish proteins. (TIF) [file pbio.1002481.s002.tif]

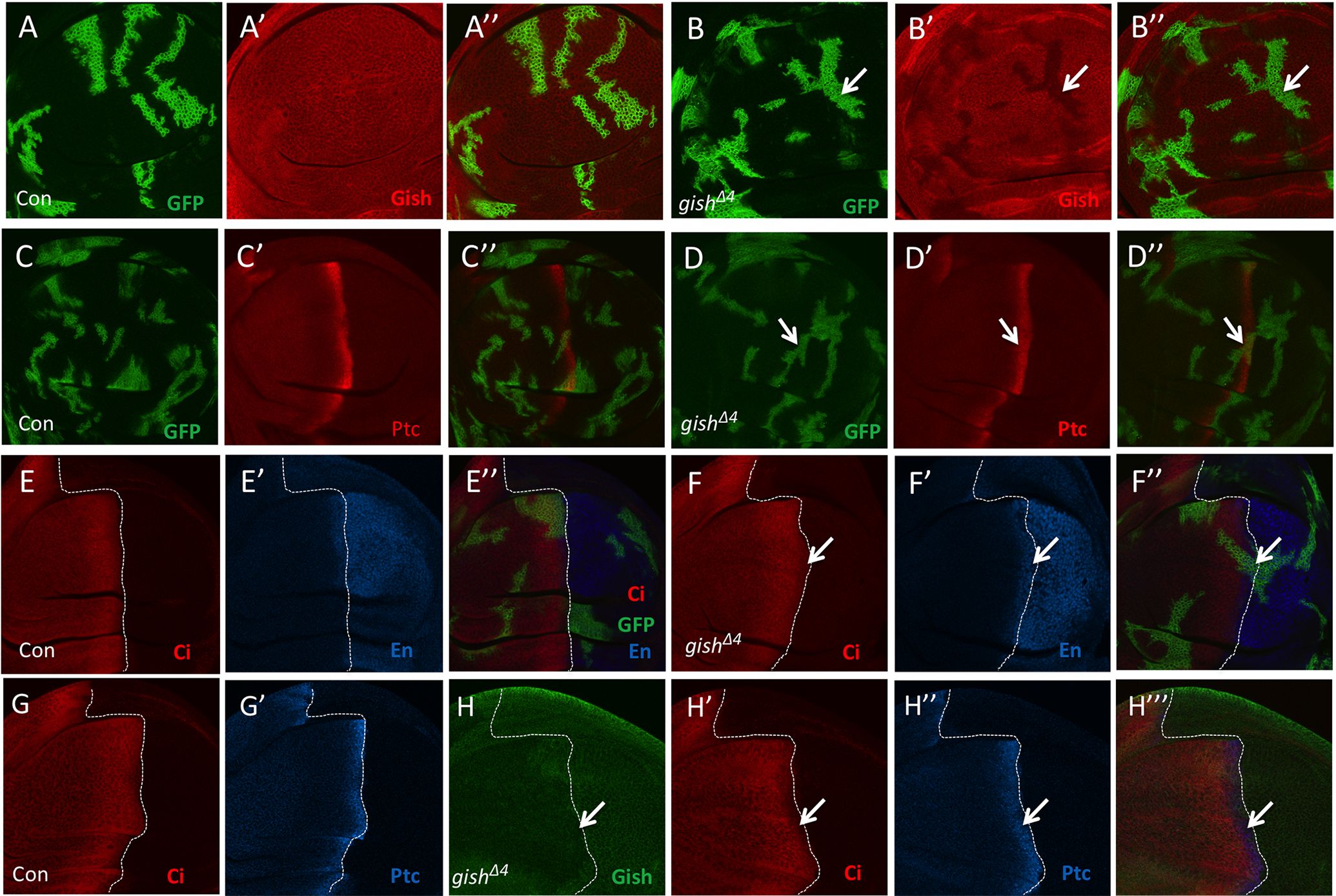

Supplement: S2 Fig — (A–F") Wing discs carrying control or gishΔ4 clones generated by the MARCM system were immunostained for GFP (green), Gish (red in A', A", B', B"), Ptc (red in C', C", D', D"), Ci (red in E, E", F, F"), and En (blue in E', E", F', F"). Clones were induced at 48–72 h AEL and marked by GFP expression. Gish expression was abolished in gishΔ4 clones (B–B"). Neither ptc nor en expression was affected in gishΔ4 clones (arrows in D–D", F–F"). (G–H‴) A control wing disc (G, G') or wing disc carrying gishΔ4 clones generated in the Minute background at 24–48 h AEL were immunostained with Ci, Ptc, and Gish antibodies. ptc expression was not affected in gishΔ4 clones marked by the lack of Gish signal (arrows in H–H‴). (TIF) [file pbio.1002481.s003.tif]

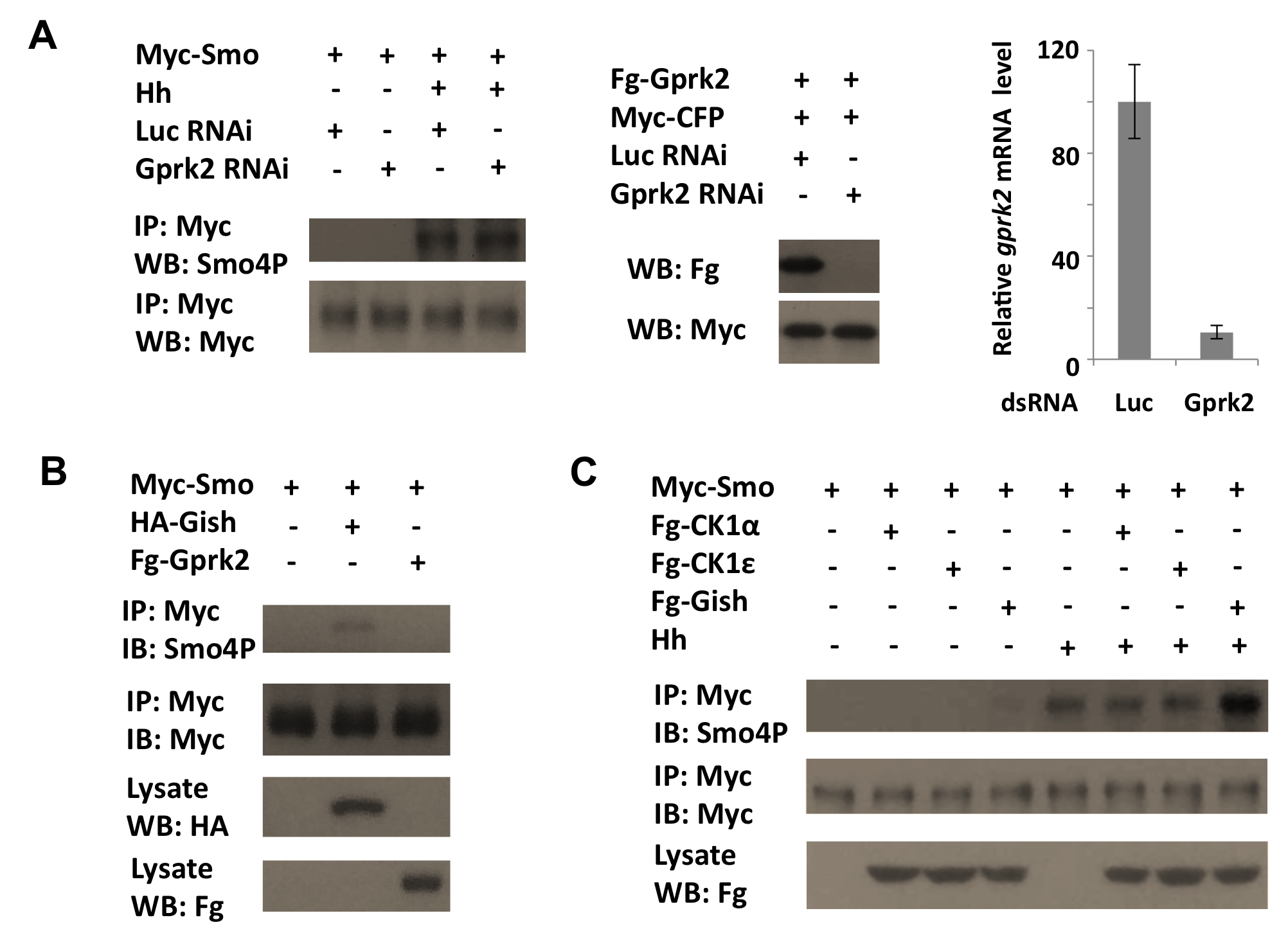

Supplement: S3 Fig — (A) S2 cells stably expressing Myc-Smo were treated with control or Gprk2 dsRNA and with or without Hh-conditioned medium, followed by immunoprecipitation and western blot analysis with the indicated antibodies. Western blot (middle panel) and RT-qPCR (right) experiments confirm Gprk2 knockdown efficiency.(B, C) Western blots of coimmunoprecipitation experiments from lysates of S2 cells transfected with Myc-Smo and the indicated CK1 or Gprk2 constructs. Cells were grown in the presence or absence of Hh stimulation for 24 h and exposed to MG132 (50 μM) for 4 h before harvesting. (TIF) [file pbio.1002481.s004.tif]

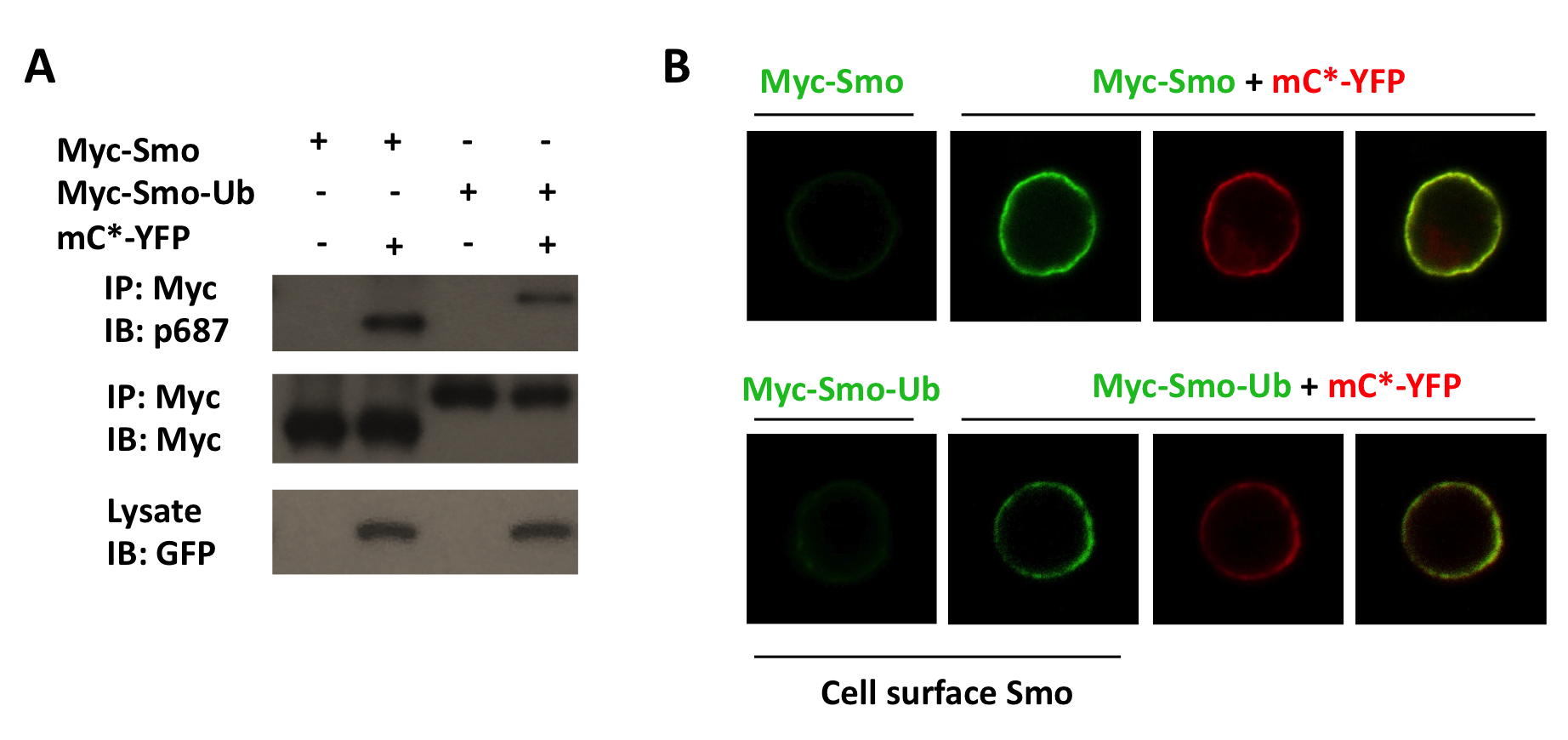

Supplement: S4 Fig — (A) Western blot of coimmunoprecipitation experiments from lysates of S2 cells transfected with Myc-Smo or Myc-Smo-Ub together with mC*-YFP (a constitutively active form of PKA). Both Myc-Smo and Myc-Smo-Ub were phosphorylated by mC* at S687.(B) S2 cells were transfected with Myc-Smo or Myc-Smo-Ub either alone or together with mC*-YFP, followed by immunostaining to visualized cell surface Myc-Smo or Myc-Smo-Ub and mC*-CFP. Coexpression with the constitutively active form of PKA resulted in cell surface accumulation of both Myc-Smo and Myc-Smo-Ub. (TIF) [file pbio.1002481.s005.tif]

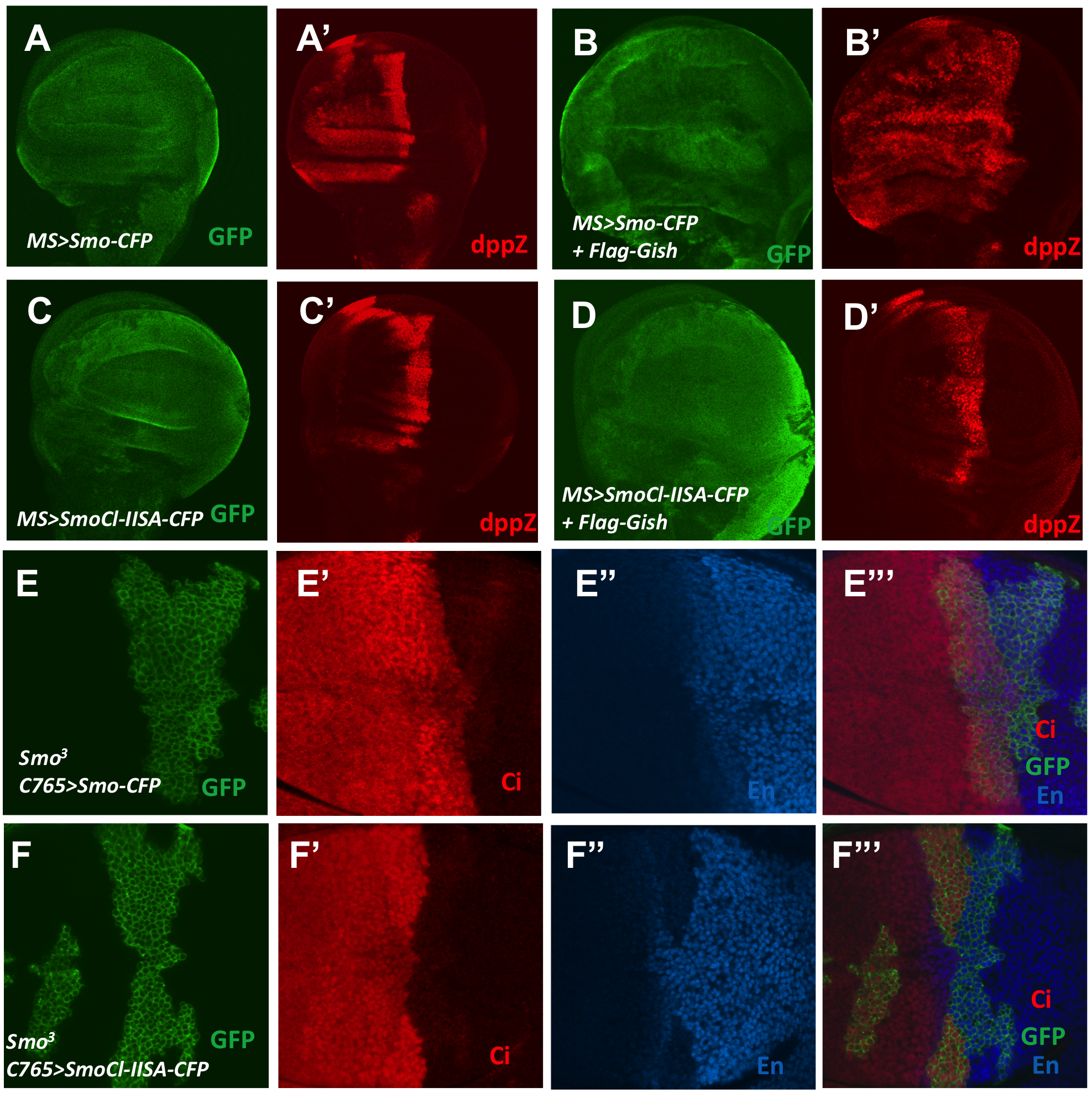

Supplement: S5 Fig — (A–D') dpp-lacZ and GFP expression in wing discs expressing the indicated Smo construct in the presence or absence Flag-Gish. Coexpression of Flag-Gish with Smo-CFP promoted its activity as indicated by more robust ectopic dpp-lacZ expression and enhanced overgrowth of the wing disc (B–B' compared with A–A'). Coexpression of Flag-Gish with SmoCL-IISA-CFP did not cause discernable change in the expression of dpp-lacZ or disc growth (D–D' compared with C–C'). (E–F‴) Wing discs carrying smo3 mutant clones expressing C765>Smo-CFP (E–E‴) or C765>SmoCL-IISA-CFP (F–F‴) were treated with 50 nM LMB for 2 h prior to immunostaining with GFP, Ci, and En antibodies. Both Smo-CFP and SmoCL-IISA-CFP promote Ci nuclear localization in smo mutant clones near the A/P boundary. (TIF) [file pbio.1002481.s006.tif]

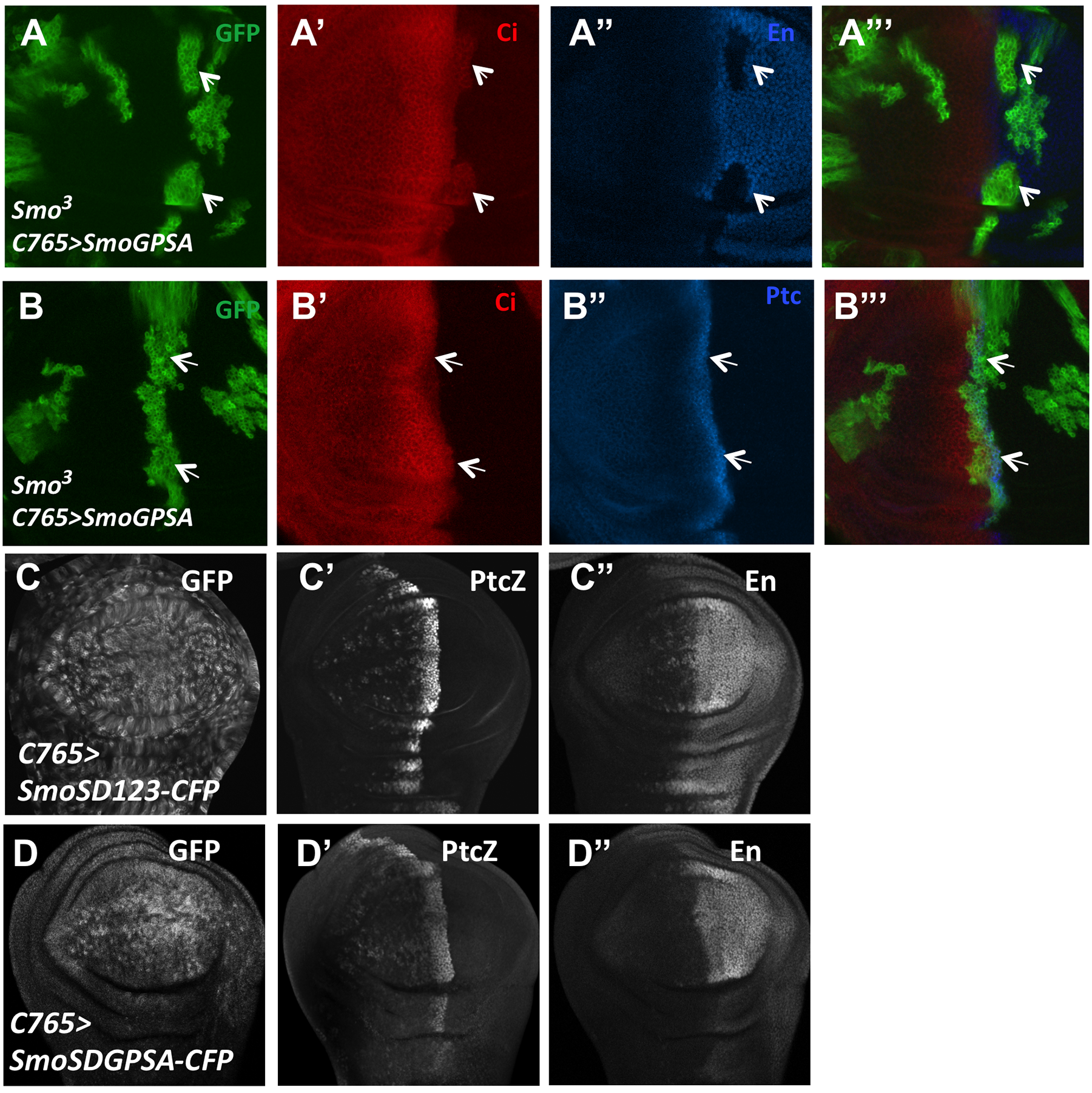

Supplement: S6 Fig — (A–B‴) Wing discs carrying smo3 mutant clones expressing C765>SmoGPSA12 were immunostained to show the expression of GFP (green), which marks the smo mutant cells, Ci (red), and En (blue in A", A‴) or Ptc (blue in B", B‴). Expression of SmoGPS1A2A with C765 rescued ptc but not en expression in smo3 mutant clone near the A-P boundary (arrowheads). (C–D'') Wing discs expressing C765>SmoSD123-CFP (C–C") or C765>SmoSDGPSA-CFP (D–D") were immunostained to show the expression of GFP, Ptc, and En. SmoSDGPSA exhibited reduced activity compared with SmoSD123. (TIF) [file pbio.1002481.s007.tif]
